# Reconstructing clonal evolution in relapsed and non-relapsed Burkitt lymphoma

**DOI:** 10.1038/s41375-020-0862-5

**Published:** 2020-05-14

**Authors:** Katrin Reutter, Sarah Sandmann, Jonas Rohde, Stephanie Müller, Marius Wöste, Tasneem Khanam, Ulf Michgehl, Wolfram Klapper, Wilhelm Wößmann, Jochen Seggewiß, Georg Lenz, Martin Dugas, Birgit Burkhardt

**Affiliations:** 1 Paediatric Hematology & Oncology, University Hospital Münster, Münster, Germany; 2 Institute of Medical Informatics, University of Münster, Münster, Germany; 3 Department of Pathology, Hematopathology Section, University Hospital Schleswig-Holstein, Kiel, Germany; 4 Paediatric Hematology & Oncology, University Hospital Hamburg, Hamburg, Germany; 5 Institute of Human Genetics, University Hospital Münster, Münster, Germany; 6 Hematology & Oncology, University Hospital Münster, Münster, Germany

**Keywords:** Genetics research, Cancer models, Cancer genetics, B-cell lymphoma

## To the Editor:

Burkitt lymphoma (BL), including its leukemic complement Burkitt leukemia (B-AL, ≥25% bone marrow infiltration), is a mature highly aggressive B-cell Non-Hodgkin Lymphoma. Current intensive short-pulse chemotherapy regimen reach event-free survival rates about 90% [1]. However, patients suffering relapse have a poor chance to survive (5-year survival rate <30%) [2].

Due to adverse prognosis and lack of knowledge on mechanisms leading to relapse, we assessed mutational status of patients with sporadic BL, comparing remission with relapse samples (for Methods see Supplementary Information, Section 1, Tables S1–S5 and Figs. S1–S5). All patients were registered in NHL-BFM data center, providing informed consent. High-coverage whole-exome sequencing (WES) analysis reveals in total 481 somatic single nucleotide variants (SNVs) and indels (see Supplementary information, Section 2.1, Tables S6–S7, Figs. S6–S9 and Supplementary Data S1 A–D; sequencing data available at NCBI Sequence Read Archive PRJNA561490). On average, primary samples from patients with relapse are characterized by a higher mutation rate compared with patients without relapse (26.4 vs. 22.6 SNVs and indels/sample; descriptive statistic, two-sample *t*-test: *p* = 0.4542). In addition, a considerably higher mutation rate can be observed for the relapse samples (47.2 SNVs and indels/sample; compared with primary with relapse: *p* = 0.2250, compared with primary without relapse: *p* = 0.1626). Pathway analyses show that genes of proliferation/cell cycle/apoptosis are most often affected by mutations in our cohort. Mutation significance analysis identifies four already known driver genes: *CCND3*, *DDX3X*, *ID3*, and *TP53* [3, 4]. All of these genes are affected differently by mutations in relapse vs. non-relapse BL. Detailed mutation analyses by lollipop plots show that for all these genes relapse patients are characterized by a different mutation profile compared with non-relapse BL. Similar results are observed with respect to copy number variants (CNVs). In total, 93 CNVs are identified by analysis of SNP array data (see Supplementary information, Sections 2.1 and 2.2.1, Figs. S10–S12 and Supplementary Data S2A). Primary samples from patients suffering relapse show on average a higher rate of CNVs compare to those from patients without relapse (7.4 vs. 1.6 CNVs/sample, *p* = 0.2159). A further increase in the average mutation rate can be detected in relapse samples (9.4 CNVs/sample; compared with primary with relapse: *p* = 0.7246, compared with primary without relapse: *p* = 0.1094). All relapse samples can be categorized as complex karyotype (>3 CNVs) [5]. It has to be noted that, additionally, every patient carries the IG-*MYC* translocation as verified by fluorescent in situ hybridization.

Since research in other hematological diseases, e.g., pediatric acute myeloid leukemia, found evidence for relapse being associated with rising subclones [6], we combined information on SNV, indel and CNV calling to reconstruct clonal evolution for all patients (see Supplementary Data S3 A–J). This is realized by analyzing the variant allele frequency (VAF) of mutations at two time points (primary and relapse) in comparison to matching germline samples. Considering previous studies on clonal evolution, e.g., Melchardt et al. [7], we noticed that nomenclature in this field currently lacks standardization. Transferring the related informatics concept of a tree to clonal evolution, we define a parent clone as an initial clone, developing from normal cells. All children developing from a parent are considered subsequent subclones (term “clone” used for parents; term “subclone” used for children). These subclones harbor all mutations of their parent clone. Siblings, i.e., parallel subclones, originate from the same parent and are considered parallel dependent subclones. They are characterized by a large set of overlapping mutations and ≥1 individual mutation each. Independent subclones are originating from different parents and are thus not expected to share any mutations. We define a clone or subclone as being dominant if no other clone or subclone—dependent or independent—accounts for >5% of the cells. Otherwise, clones and subclones are considered co-existent. Because mutations are propagated from parent clones to subclones, we assume that mutations with a higher VAF in dependent subclones occurred at an earlier point in time.

**Fig. 1:**
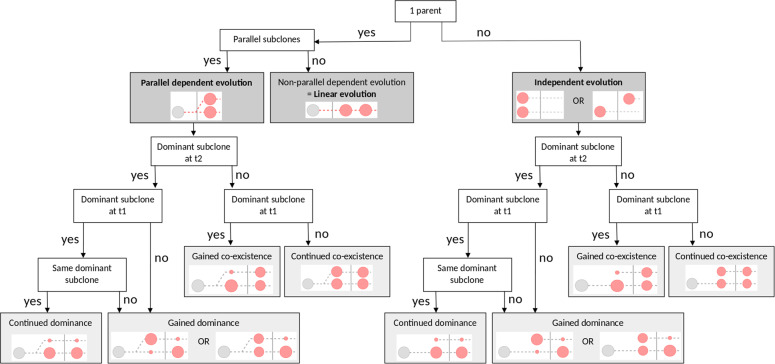
Categorization of clonal evolution. The decision tree visualizes categorization of clonal evolution comparing two time points, considering up to two parallel subclones. We propose three main categories (dark gray) and up to four subcategories (light gray). Schematic figures visualize the essential characteristics of each main- and subcategory (red).

To systematically categorize clonal evolution, comparing two time points, we propose the following three main categories: independent evolution, parallel dependent evolution and non-parallel dependent evolution, i.e., linear evolution. Parallel dependent and independent evolution can be further distinguished by four subcategories: gained dominance, continued dominance, gained co-existence, and continued co-existence. Figure 1 provides a decision tree, illustrating how these categories are assigned. Transferring this systematic nomenclature to the set of relapse patients analyzed in this study, we observe different types of clonal evolution (see Supplementary Information, Section 2.3.1, Table S8).

**Fig. 2:**
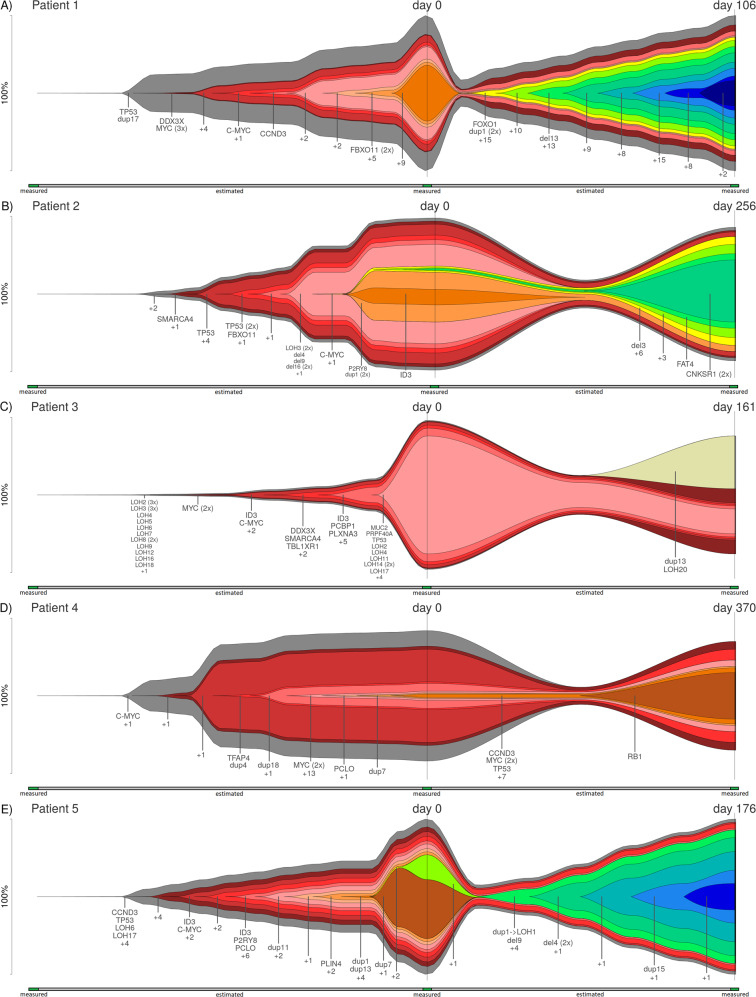
Clonal evolution of relapsing patients. Measurements for three time points are available: germline, primary, and relapse. Clonal evolution is derived comparing two time points (primary and relapse) to the germline sample. The percentage of affected cells is provided on the *y*-axis. C-MYC indicates a C-*MYC* rearrangement. **a** Patient 1: at primary diagnosis (day 0), subclone no. 9 (orange; 36% of the cells) is dominant. In progress, this subclone disappears and a new dependent subclone (no. 10—yellow; originating from subclone no. 8—intermediate-orange) expands. Several additional mutations, inter alia (i.a.), *FOXO1*, dup1 and del 13, are acquired up to day 106 of relapse (subclones no. 11–17—green to dark blue). **b** Patient 2: At day 0, there are 2 parallel, dependent subclones: Subclones no. 8 and 9 (orange; dup1, *P2RY8* and *ID3*) are dominant (~32 and 10% of the cells) over subclone no. 10 (yellow; 4%; i.a. del3). At relapse (day 256), the subclones no. 8 and 9 have lost their dominance in favor of subclone no. 10 and its successive subclones no. 11–13 (green; 4 vs. 70%). **c** Patient 3: Subclone no. 6 (light red) is dominant at day 0 (76% of the cells; i.a. mutations in *MUC2*, *PRPF40A* and *TP53*). However, it has lost dominance at relapse (day 161; 16%) and co-exists with newly emerged, dependent subclone no. 7 (beige; 34%; dup13 and LOH20). **d** Patient 4: All mutations are successively acquired; linear evolution can be observed. Only one relapse-specific mutation is detected: a frameshift mutation in *RB1* (subclone no. 10—brown). **e** Patient 5: Two dependent subclones co-exist at day 0. Subclone no. 10 (brown; 38% of the cells at time point 1; dup7 and *FIGN*) disappears. Subclones no. 11 and 12 (yellow and light green; 16% of the cells at time point 1, 86% at time point 2; *KIZ*, *SF3B1,* and *FMN1*) gains 13 additional mutations (including 5 CNVs), survives therapy and finally leads to relapse (day 176).

Our observations indicate parallel dependent evolution in a majority of samples. Three patients (see Fig. 2a, b, e) are characterized by gained dominance of the dependent subclones, and one (see Fig. 2c) by gained co-existence of the dependent subclones. Their common feature—existence of parallel subclones—increases genetic diversity, which might enable fast progression, therapy resistance, and failure. All patients feature an advanced stage phenotype and likely deficient *TP53* due to ≥2 mutations (patient 1: SNV and CNV; patient 2: 3 SNVs; patient 3: insertion and CNV; patient 5: SNV and CNV). Previous studies reported *TP53* deficiency combined with drug resistance to BL therapy, associated with shorter progression-free survival. *MYC-ARF-TP53* axis is reported to be the primary deregulated one in BL [5, 8].

Linear evolution, as reported by Aukema et al. [9] for patients with relapsing BL, can only be observed for one patient (see Fig. 2d). According to Melchardt et al. [7], linear evolution with only minimal change in relapse may indicate a pre-programmed resistance. Interestingly, this patient features the longest time to first relapse (370 days; other patients: 106–256 days). Only a single relapse-specific mutation can be detected (frameshift mutation in *RB1*). However, data indicate *TP53* deficiency due to two mutations like in all relapse cases: the gene is affected by an SNV and a low-frequency CNV (see Supplementary information, Section 2.2.1, and Fig. S12).

Concluding, our findings indicate that relapse patients are characterized by complex karyotype and likely deficient *TP53* resulting from presence of ≥2 mutations. A majority of patients features non-linear clonal evolution, characterized by new aggressive subclones. These subclones are—with low frequencies—often already detectable at primary diagnosis. Linear evolution may increase the time to relapse. This was also reported for pediatric acute myeloid leukemia [6].

Different from other studies investigating clonal evolution, e.g., da Silva-Coelho et al. [10] studying myelodysplastic syndromes (MDS), BL cannot be followed over many years due to the aggressive nature, which quickly leads to death. Due to the presence of only two time points, we developed a bioinformatic approach to estimate the development of clones over time as well as the effect of therapy. This approach allows for reconstructing a valid clonal evolution despite few measured time points, reveals new insights into the genetic development of BL and outperforms currently available software for automatic reconstruction of clonal evolution (see Supplementary information, Section 2.3.2, Figs. S13–S14, Section 2.3.3, Fig. S15 and Section 2.3.4, Tables S9–S10).

SNP arrays could be an option to analyze CNVs in clinical routine, since normal karyotyping is often not possible in BL due to the fragility of the cells in culture. Furthermore, therapy course should be linked to clonal evolution. In MDS, similar analyses have shown that therapy may create an evolutionary selection of more complex, but also unrelated clones [10]. In addition, the mutation status of *TP53* in terms of *TP53* deficiency should be considered. Research on related diseases as well as other cohorts of BL support this suggestion: In acute myeloid leukemia *TP53* deficiency permits chromosomal complexity [11]. Also it switches T-cell lymphoma development into aggressive one [12]. In childhood acute lymphoblastic leukemia and in BL an association of *TP53* alterations and inferior outcome was found [5, 13]. Detailed analysis of clonal evolution in our study suggests that relapse is not only associated with alterations in *TP53*, but with *TP53* deficiency. All relapsing patients are characterized by likely deficient *TP53*, caused by damaging point mutations and CNVs. In our study, tumors of patients without relapse do not harbor mutations in *TP53* other than R248Q with a VAF < 50%. This indicates a heterozygous variant with still functioning *TP53*. Considering other cohorts of patients with BL similar results can be observed. Panea et al. [14] analyzed 60 cases of non-relapsing sporadic BL using whole-genome sequencing, which allows for analysis of small as well as large mutations. Five patients feature mutations in *TP53* affecting the exon or the splice site. However, a maximum of only one mutation per patient can be observed. It will be the next step to consider clonal evolution and *TP53* deficiency in a larger cohort of BL patients with relapse. Our analyses emphasize the importance of an extensive genetic characterization, including *TP53*, at initial time point. As practical implementation we suggest CNV analyses plus sequencing of a Burkitt specific target panel, better even WES.

## Supplementary information

Supplementary Information

SupplementaryData_1.xlsx

SupplementaryData_2.xlsx

SupplementaryData_3.xlsx
