# Publisher Correction: Reconstructing clonal evolution in relapsed and non-relapsed Burkitt lymphoma

**DOI:** 10.1038/s41375-021-01359-2

**Published:** 2021-11-16

**Authors:** Katrin Reutter, Sarah Sandmann, Jonas Rohde, Stephanie Müller, Marius Wöste, Tasneem Khanam, Ulf Michgehl, Wolfram Klapper, Wilhelm Wößmann, Jochen Seggewiß, Georg Lenz, Martin Dugas, Birgit Burkhardt

**Affiliations:** 1 Paediatric Hematology & Oncology, University Hospital Münster, Münster, Germany; 2 Institute of Medical Informatics, University of Münster, Münster, Germany; 3 Department of Pathology, Hematopathology Section, University Hospital Schleswig-Holstein, Kiel, Germany; 4 Paediatric Hematology & Oncology, University Hospital Hamburg, Hamburg, Germany; 5 Institute of Human Genetics, University Hospital Münster, Münster, Germany; 6 Hematology & Oncology, University Hospital Münster, Münster, Germany

**Keywords:** Genetics research, Cancer models, Cancer genetics, B-cell lymphoma

Correction to: *Leukemia* 10.1038/s41375-020-0862-5

The article “Reconstructing clonal evolution in relapsed and non-relapsed Burkitt lymphoma”, written by Katrin Reutter, Sarah Sandmann, Jonas Rohde, Stephanie Müller, Marius Wöste, Tasneem Khanam, Ulf Michgehl, Wolfram Klapper, Wilhelm Wößmann, Jochen Seggewiß, Georg Lenz, Martin Dugas and Birgit Burkhardt was originally published Online First without Open Access. After publication in volume 35, pages 639–643, the author decided to opt for Open Choice and to make the article an Open Access publication. Therefore, the copyright of the article has been changed to © The Author(s) 2020 and the article is forthwith distributed under the terms of the Creative Commons Attribution.

