# Supplementary material for: Reconstructing clonal evolution in relapsed and non-relapsed Burkitt lymphoma
Source: Leukemia. 2020 May 14;35(2):639–43. doi: 10.1038/s41375-020-0862-5 (PMC8318876; doi:10.1038/s41375-020-0862-5)
Supplement: Supplementary file 1 — Supplementary Information [file 41375_2020_862_MOESM1_ESM.pdf]

Supplement: Reconstructing Clonal Evolution in Relapsed and Non-Relapsed Burkitt Lymphoma

Contents

1 Supplementary Methods **6**
1.1 Patients and samples . . . 6
1.2 DNA isolation and amplification . . . 6
1.3 Whole-exome sequencing . . . 6
1.3.1 Driver genes . . . 8
1.3.2 Literature research . . . 8
1.3.3 Pathway information . . . 9
1.3.4 Data availability . . . 9
1.4 Targeted sequencing . . . 9
1.5 Sanger sequencing . . . 9
1.6 SNP array . . . 9
1.7 Fluorescent *in situ* hybridization . . . 10
1.8 Categorizing clonal evolution . . . 10
1.9 Reconstructing clonal evolution . . . 11
1.9.1 Estimating cell fractions for CNVs . . . 12
1.9.2 Estimating development of clones over time . . . 13
1.9.3 Estimating effect of therapy . . . 14
1.9.4 Automatic approaches . . . 15

2 Supplementary Results **17**
2.1 SNV and indel calling . . . 17
2.2 CNV calling . . . 19
2.2.1 CNV calls on chromosome 17 . . . 22
2.3 Clonal evolution . . . 23
2.3.1 Applied categorization . . . 23
2.3.2 Proof of concept: exemplary application of our estimation approach . . . 24
2.3.3 Clonal evolution without estimated time points . . . 26
2.3.4 Automatically reconstructed clonal evolution . . . 26
2.4 Supplementary Data . . . 29

List of Figures

S1 **Categorizing clonal evolution.** Categories for clonal evolution at two time points. Three main categories can be distinguished: independent evolution, parallel dependent evolution and non-parallel dependent evolution, i.e. linear evolution. 11

S2 **Relation between VAF of a polymorphism and fraction of cells affected by CNV.** Copy number variations lead to a change in VAF of heterozygous polymorphisms. If the fraction of cells affected by CNV is unknown, the observed change in VAF can be used for estimation (deletions: black; duplications: red). . 12

S3 **Exemplary clonal evolution without estimated time points.** Example for linear clonal evolution, including 1 clone and 3 subclones with measurements at 2 time points. Every (sub)clone is characterized by one mutation each. A) Clonal evolution. B) Percentage of cells with mutations. 13

S4 **Exemplary clonal evolution with estimated time points.** Example for linear clonal evolution, including 1 clone and 3 subclones with measurements at 2 time points and estimated cells with mutations at additional time points. Every (sub)clone is characterized by one mutation each. A) Clonal evolution with 2 estimated time points. B) Clonal evolution with 3 estimated time points. C) Percentage of cells with mutations, estimated time points are marked with an asterisk. 15

S5 **Exemplary clonal evolution with estimated time points and estimated therapy effect.** Example for linear clonal evolution, including 1 clone and 3 subclones with measurements at 2 time points and estimated cells with mutations at 4 additional time points. Every (sub)clone is characterized by one mutation each. A) Clonal evolution. B) Percentage of cells with mutations, estimated time points are marked with an asterisk. 16

S6 **Summarized SNV and indel calls.** Distribution of SNVs and indels over all patients (orange: primary specific; green: relapse specific; blue: shared mutations). 17

S7 **Oncoplot of the most frequently mutated genes.** Graphical overview of the most frequently mutated genes, including SNVs, indels and CNVs. Genes affected by mutations are clustered according to affected pathway. 17

S8 **Lollipop plots for significantly mutated genes.** Mutations detected in A) *CCND3*, B) *DDX3X*, C) *ID3*, D) *TP53* in relapse and non-relapse patients. All genes have been identified as significant driver genes. 20

S9 ***In silico* evaluation of mutations.** Provean prediction scores for somatic mutations detected in primary and relapse samples (default cutoff for deleterious < -2.5). Prediction scores for mutations in TP53 are marked in red. Missing prediction scores (NA) result from frameshift mutations and splice site mutations. 21

S10 **Summarized CNV calls.** Distribution of CNVs over all patients (orange: primary specific; green: relapse specific; blue: shared mutations). 21

S11 **Detailed CNV calls.** CNVs detected for every patient (blue: deletion; yellow: LOH; red: duplication). 22

S12 **CNVs on chromosome 17.** SNP array data showing chromosome 17 (plots visualizing B allele frequency and log R ratio, generated by Illumina GenomeStudio 2.0) for patients 4 and 5 (primary and relapse) as well as patients 6 to 10 (primary). The red lines mark the position of *TP53*. 23

S13 **Estimated and real clonal evolution in MDS.** Clonal evolution for MDS patients UPN03, UPN06, UPN07 and UPN11. A, C, E, G) Evaluating all measured time points. B, D, F, H) Evaluating the first and last measured time points and adding additional estimated time points. 25

S14 **Estimated and real clonal evolution in MDS including therapy effect.** Clonal evolution for MDS patients UPN01, UPN08 and UPN09. A, C, E) Evaluating all measured time points. B, D, F) Evaluating the first and last measured time points and adding additional estimated time points. 26
S15 **Alternative clonal evolution plots for relapse patients 1-5.** The plots show just the 2 measured time points and no estimated development in between. A) Patient 1. B) Patient 2. C) Patient 3. D) Patient 4. E) Patient 5. 27

List of Tables

S1 **Clinical characteristics.** Information on clinical characteristics of the cohort (BM, bone marrow; CNS, central nervous system; F, female; LDH, lactate dehydrogenase highest increase; M, male). 6

S2 **Primary- and relapse manifestation.** Information on primary- and relapse manifestation of the cohort (BM, bone marrow; CNS, central nervous system; LN, lymph node; PE, pleura/pleural effusion). 7

S3 **Therapy complications and modifications.** Information on therapy complications and modifications of the cohort (ICU, intensive care unit; TLS, tumor lysis syndrome). 7

S4 **WES data characteristics.** Sequencing data characteristics of all WES samples: Germline samples 1 to 10 (G_1 to G_10), primary samples 1 to 10 (P_1 to P_10) and relapse samples 1 to 5 (R_1 to R_5). 8

S5 **Targeted sequencing data characteristics.** Sequencing data characteristics of all targeted sequencing samples, analyzed for validation: Germline samples 1 to 10 (G_1 to G_10), primary samples 1 to 10 (P_1 to P_10; including 4 re-sequenced samples) and relapse samples 1 to 5 (R_1 to R_5). 10

S6 **Gene enrichment analysis.** KEGG pathways reported from gene enrichment analysis with DAVID. No significant results can be observed when considering the adjusted p value. 18

S7 **MutSigCV output.** MutSigCV output-file sig_genes.txt including the top-10 significant genes (N_nonsilent, number of covered sequenced bases containing non-silent mutations; N_silent, number of covered sequenced bases containing silent mutations; N_noncoding, number of covered sequenced bases containing noncoding mutations; n_nonsilent, number of non-silent mutations; n_silent, number of silent mutations; n_noncoding, number of noncoding mutations; nnei, number of neighboring genes; x, number of mutated bases in neighboring genes; X total number of bases related to neighboring genes; p, p-value; q, q-value, i.e. adjusted p-value). q values indicate that 4 genes are significant with $\alpha < 0.05$: *CCND3*, *DDX3X*, *ID3* and *TP53*. 18

S8 **Applied categorization for clonal evolution.** Main categories and subcategories for clonal evolution at two time points. Information on our samples with relapse (Patients 1-5) and samples analyzed by da Silva-Coelho *et al.* 2017 [1] (UPNs) were added to the matching main- and subcategories. 24

S9 **Clonal evolution with PyClone and ClonEvol.** Automatically reconstructed clonal evolution, using PyClone in combination with ClonEvol, in comparison to manually reconstructed clonal evolution. 27

S10 **Clonal evolution with SciClone and ClonEvol.** Automatically reconstructed clonal evolution, using SciClone in combination with ClonEvol, in comparison to manually reconstructed clonal evolution. Application of ClonEvol is only possible in the presence of >1 time point, i.e. not for the non-relapse samples. 28

1 Supplementary Methods

1.1 Patients and samples

We collected material from 10 patients with confirmed diagnosis of Burkitt lymphoma or Burkitt leukemia (BL) (≥25% bone marrow infiltration). All patients are characterized by C-*MYC* rearrangement. Average age at diagnosis is 8.2 years (range 4-15). All patients were registered in NHL-BFM data center and received risk-adapted standard protocol treatment stratified according to stage and lactate dehydrogenase (LDH) [2].

For all patients we collected a germline sample (bone marrow or peripheral blood without blast infiltration) and a sample at primary point of diagnosis (native tumor, bone marrow with blasts ≥75% or malignant effusion material). For 5 relapse patients (the term "relapse" covers disease progression and relapse), an additional matching sample at the point of relapse was collected. All relapse patients suffered death of disease, while all non-relapse patients are still alive (follow-up: 48-97 months). Patient characteristics are summarized in tables S1 to S3.

Table S1: Clinical characteristics. Information on clinical characteristics of the cohort (BM, bone marrow; CNS, central nervous system; F, female; LDH, lactate dehydrogenase highest increase; M, male).

Patient	Sex	Age (years)	BM (% blasts)	CNS	LDH (U/l)	Risk group	Relapse (days from diagnosis)
1	M	6	97%	yes	25,800	R4, CNS+	106
2	F	7	no	no	2,311	R4	256
3	M	8	12%	no	631	R3	151
4	F	6	96%	yes	6,564	R4, CNS+	370
5	F	15	97%	no	19,328	R4	176
6	M	10	no	no	3,574	R4	-
7	M	5	no	no	612	R3	-
8	M	4	99%	yes	858	R4, CNS+	-
9	M	11	91%	no	1,761	R4	-
10	M	10	92%	no	387	R3	-

1.2 DNA isolation and amplification

Genomic DNA was extracted using DNeasy Blood and Tissue Kit (07/2006: Animal Blood-Spin Column Protocol) according to manufactures guidelines.

1.3 Whole-exome sequencing

Whole-exome sequencing (WES) was performed on 10 germline samples, 10 primary tumor samples and 5 relapse samples. The genomic library was prepared using SureSelect Human All Exon V6 + Cosmic (Agilent). Illumina NextSeq (2x150 bp) was used for sequencing.

Germline samples are characterized by an average coverage of 44.21x. For primary samples, we observe average coverage of 277.28x, for relapse samples average coverage is 298.79x. Detailed sequencing data characteristics are provided in table S4.

Sequencing data is aligned to the human reference genome GRCh37 using BWA mem [3] (default options for paired-end data). Duplicate reads are removed using Picardtools (http://broadinstitute.github.io/picard/).

Somatic single nucleotide variants (SNVs) are detected using SomaticSniper [4], applying a high-confidence filter (mapping quality >40, somatic score >40). Somatic insertions/deletions (indels) are detected using Strelka [5] with default quality filter settings.

To improve sensitivity and specificity of our results, we apply additional filter- and analysis steps. 1) All variants with evidence in any germline sample are removed (minimum depth=10,

Table S2: Primary- and relapse manifestation. Information on primary- and relapse manifestation of the cohort (BM, bone marrow; CNS, central nervous system; LN, lymph node; PE, pleura/pleural effusion).

Patient	Primary manifestations	Relapse manifestations
1	LN cervical + nuchal, spleen, liver, LN inguinal, BM, CNS	CNS, BM
2	PE, abdominal LN, bowel, ovary (bilateral), ascites	PE, mediastinum, liver, spleen, abdominal LN, pancreas, kidney (bilateral), BM
3	Bowel, abdominal LN, peripheral LN, ascites, BM	PE, bowel
4	LN submandibular, BM, CNS	BM, CNS, PE, mandible
5	LN cervical, PE, liver, abdominal LN, ascites, BM	Right mammary, LN axillar
6	Mediastinum, pericardium/pericardial effusion, PE, lung, peripheral LN, ascites	-
7	LN supraclavicular, multifocal cervical spine, PE, abdominal LN, liver, bowel, LN inguinal, ascites, mediastinum	-
8	Mucosal and osseous lesions of midface and jaw, LN cervical, liver, kidney (bilateral), spleen, abdominal LN, peripheral LN, BM, bone (humerus), CNS, epidural	-
9	Liver, spleen, BM	-
10	LN cervical, liver, stomach, kidney (bilateral), BM	-

Table S3: Therapy complications and modifications. Information on therapy complications and modifications of the cohort (ICU, intensive care unit; TLS, tumor lysis syndrome).

Patient	Complication	Modification
1	Prolonged methotrexate excretion, septicaemia	-
2	3rd course: septecaemia, ICU	-
	4th course: norovirus gastroenteritis	-
	5th course: clostridium difficile enteritis	-
	6th course: norovirus gastroenteritis	-
3	-	-
4	Steroid associated diabetes requiring insulin substitution	-
5	Capillary leak syndrome, ICU	-
	Allergic reaction after etopophos, ICU	Reduction of treatment (etoposide, anthracyclines, 6th course)
6	TLS	Reduction of dexamethason
7	-	-
8	Pneumonia with need of oxygen	-
9	-	-
10	-	-

minimum number of alternate reads=2, minimum variant allele frequency VAF=0.02). Thus, common germline calls that have been reported due to low coverage in the matching germline samples can be identified. 2) Additional variant calling is performed using VarScan [6] and MuTect2 [7]. Both tools allow for matched sample analysis. We use VarScan version 2.3.9 (command `somatic`) and GATK version 4.0.4.0 (command `Mutect2`). Results are compared to and combined with the SomaticSniper/Strelka output to increase sensitivity. 3) Additional variant calling is performed using appreci8 [8] (minimum depth=10, minimum number of alternate reads=3, minimum VAF=0.01). Different from SomaticSniper, Strelka, VarScan and MuTect2, appreci8 analyzes non-matched samples. Comparing variant calling results from a totally different approach allows us to identify additional false positive and false negative calls. Furthermore,

Table S4: WES data characteristics. Sequencing data characteristics of all WES samples: Germline samples 1 to 10 (G_1 to G_10), primary samples 1 to 10 (P_1 to P_10) and relapse samples 1 to 5 (R_1 to R_5).

Sample	Total reads	Mapped reads		Uniquely mapped		Proper read pairs		Mean coverage	Coverage ≥1x	Coverage ≥10x	Coverage ≥100x
G_1	33924649	33826810	99.71%	32587525	96.34%	33535380	99.14%	50.94	98.21%	93.73%	8.71%
P_1	190077305	188540410	99.19%	181628464	96.33%	185589527	98.43%	302.81	98.43%	95.92%	65.42%
R_1	244294627	242665199	99.33%	235617268	97.10%	240034627	98.92%	220.86	98.55%	97.90%	83.07%
G_2	22922803	22815965	99.53%	22079977	96.77%	22653170	99.29%	35.79	97.77%	90.36%	1.60%
P_2	217765806	215924772	99.15%	208381918	96.51%	213397736	98.83%	320.69	98.41%	97.94%	92.41%
R_2	229448734	227292017	99.06%	219074079	96.38%	224370318	98.71%	350.84	98.38%	97.87%	87.29%
G_3	18877753	18828765	99.74%	18314557	97.27%	18675464	99.19%	33.42	97.26%	83.70%	2.60%
P_3	135965818	135265832	99.49%	131266796	97.04%	132976158	98.31%	232.45	98.45%	97.59%	81.43%
R_3	152556506	151632065	99.39%	146955406	96.92%	149567944	98.64%	263.31	98.41%	97.61%	84.13%
G_4	34252202	34127342	99.64%	33193347	97.26%	33842458	99.17%	61.11	97.87%	92.29%	18.22%
P_4	111911873	111325704	99.48%	108061669	97.07%	110557842	99.31%	192.07	98.26%	97.08%	72.38%
R_4	211843837	209918389	99.09%	203278075	96.84%	207619903	98.91%	359.07	98.39%	97.72%	89.09%
G_5	21704291	21623884	99.63%	21055811	97.37%	21431187	99.11%	38.76	97.38%	87.11%	4.45%
P_5	178135744	176750765	99.22%	171540786	97.05%	174180557	98.55%	311.01	98.19%	96.48%	74.83%
R_5	176011199	174469280	99.12%	169175015	96.97%	171838123	98.49%	299.85	98.34%	97.64%	87.30%
G_6	24751043	24658238	99.63%	23818779	96.60%	24414148	99.01%	39.00	97.89%	91.04%	2.75%
P_6	204554798	203100320	99.29%	196146187	96.58%	201326525	99.13%	309.73	98.56%	98.05%	91.25%
G_7	20935304	20844857	99.57%	20310719	97.44%	20686789	99.24%	36.82	97.61%	87.17%	3.23%
P_7	133761957	132838692	99.31%	129091841	97.18%	131543340	99.02%	228.69	98.48%	97.61%	80.49%
G_8	22037001	21979126	99.74%	21380791	97.28%	21795214	99.16%	39.36	97.52%	87.15%	5.04%
P_8	173541325	172472573	99.38%	167206384	96.95%	170288519	98.73%	295.16	98.51%	97.72%	85.30%
G_9	34187182	34057409	99.62%	33178377	97.42%	33827492	99.32%	60.55	98.00%	93.38%	16.79%
P_9	155862006	154835356	99.34%	150180987	96.99%	153127883	98.90%	271.15	98.43%	97.70%	85.14%
G_10	26019052	25937298	99.69%	25251313	97.36%	25670635	98.97%	46.32	97.75%	90.71%	7.71%
P_10	179103411	177770296	99.26%	172319617	96.93%	175757287	98.87%	309.01	98.41%	97.72%	87.23%

germline samples can be analyzed for possible contamination by tumor cells (for which no evidence was found). 4) Variants called only in one sample – primary or relapse – are re-evaluated. Coverage statistics are determined using bam-readcount (https://github.com/genome/bam-readcount) to investigate presence of the variant at very low VAF at one of the two time points we consider.

All calls are annotated with SnpEff [9] using annotations based on RefSeq (hg19). We exclude all calls annotated as 3_prime_UTR_variant, 5_prime_UTR_variant, downstream_gene_variant, upstream_gene_variant, intergenic_region, intron_variant, non_ coding_exon_variant and splice_region_variant+intron_variant.

All remaining calls are manually investigated using the IGV [10]. Variant call characteristics, involving the type of mutation, VAF, coverage, base quality and database information (ESP6500 http://evs.gs.washington.edu/EVS/, 1000 Genomes [11], ExAC [12], dbSNP [13], ClinVar [14] and Cosmic [15]), are evaluated. Furthermore, exemplary validation experiments are performed, involving targeted- as well as Sanger sequencing [16].

1.3.1 Driver genes

To determine likely driver genes, variant calling results are analyzed with MutSigCV_1.41 [17] using default parameters (genome reference sequence: chr_files_hg19.zip; MutSigCV for MAF file only). As only one mutation is detected affecting UTR (patient 5 relapse: chr6:56,846,532 C>T; 5'UTR premature start codon gain variant; *BEND6*), this variant had to be excluded from analysis with MutSig.

1.3.2 Literature research

For an in-depth analysis of every patients' mutation profile and clonal evolution, we perform literature research of every mutated gene identified by WES. "GeneCards" provides information on common aliases, an overview of known gene functions, linked pathways and related diseases [18]. Based on this information, we perform PubMed search, using the gene name and its aliases. Search is restricted by adding "neoplasm OR cancer OR lymphoma OR leukemia OR relapse OR progression" to the search string.

1.3.3 Pathway information

For genes mutated in at least 2 samples (≥20%), we considered pathway information. We upload data to Ingenuity pathway analysis (IPA; QIAGEN Inc., https://www.qiagenbioinformatics.com/products/ingenuity-pathway-analysis). Additionally, we use the R package "PaxtoolsR" version 1.16.0 [19] to add information on pathways based on KEGG, Panther, PathwayCommons and Reactome. Eighteen genes show mutations in at least 2 samples. However, 6 out of 18 do not feature any pathway information according to the described sources. Therefore, we perform additional manual search using GeneCards [18].

In a majority of cases, pathway information differed between the different sources. Furthermore, for many genes, more than one pathway was reported. Therefore, we manually combined all resulting pathway information and sorted the mutated genes into 6 functional categories: proliferation/cell cycle/apoptosis, epigenetics, gene expression, metabolism, signal transduction, and axon guidance.

1.3.4 Data availability

Sequencing data of all 10 patients have been deposited into the NCBI Sequence Read Archive (PRJNA561490). All remaining data are available within the article and Supplementary Data files (S1 A-D, S2 A and S3 A-J).

1.4 Targeted sequencing

Targeted sequencing was performed on all samples (10 germline, 10 primary, 5 relapse; independent re-sequencing was performed for 4 primary samples). The genomic library was prepared using Nextera Rapid Capture Custom Enrichment Kit. An in-house panel covering 132 genes (168,649 bp) – known or assumed to play an important role in Burkitt lymphoma – was used for sequencing on Illumina MiSeq (2x150 bp).

Average coverage of the germline samples is 674.96x. For primary samples, we observe average coverage of 806.15x, for relapse samples average coverage is 628.02x. Detailed sequencing data characteristics are provided in table S5.

Sequencing data is aligned to the human reference genome GRCh37 using BWA mem (default options for paired-end data). appreci8 is used for variant calling (default parameters for targeted sequencing data: minimum depth=50, minimum number of alternate reads=20, minimum VAF=0.01).

Altogether, 95 variants called in WES data can be confirmed by targeted sequencing data. Furthermore, 11 variant calls previously classified as likely artifacts are never called in targeted sequencing data. Detailed analyses reveal that they cannot or only at very low VAFs be observed in targeted sequencing data. Thus, their categorization can equally be confirmed. Detailed information on validation results is available in Supplementary Data 1 B-D.

1.5 Sanger sequencing

Sanger sequencing was performed to validate exemplary mutations in *CCND3*, *ID3* and *TP53* called in primary tumor samples. Altogether, 4 mutations in *CCND3*, 13 mutations in *ID3* and 3 mutations in *TP53* were validated. Detailed information on validation results is available in Supplementary Data 1 B-D.

1.6 SNP array

SNP array analysis was performed for the 10 primary and 5 relapse samples. For analysis, we used Infinium OmniExpressExome-8v1.3 kit.

Table S5: Targeted sequencing data characteristics. Sequencing data characteristics of all targeted sequencing samples, analyzed for validation: Germline samples 1 to 10 (G_1 to G_10), primary samples 1 to 10 (P_1 to P_10; including 4 re-sequenced samples) and relapse samples 1 to 5 (R_1 to R_5).

Sample	Total reads	Mapped reads		Uniquely mapped		Proper read pairs		Mean coverage	Coverage $\geq$1x	Coverage $\geq$10x	Coverage $\geq$100x
G_1	1469047	1467093	99.87%	1455693	99.22%	1455789	99.23%	731.74	99.42%	98.64%	95.27%
P_1.1	2851977	2847613	99.85%	2828748	99.34%	2801462	98.38%	1418.26	99.94%	99.61%	98.74%
P_1.2	1543630	1542180	99.91%	1529900	99.20%	1529630	99.19%	732.72	99.88%	99.54%	97.77%
R_1	1119909	1118249	99.85%	1105233	98.84%	1109268	99.20%	539.97	99.52%	98.24%	90.58%
G_2	1098018	1096422	99.85%	1088046	99.24%	1088806	99.31%	507.34	99.39%	98.73%	93.96%
P_2	2430041	2426452	99.85%	2407562	99.22%	2398873	98.86%	1210.75	99.81%	99.43%	98.25%
R_2	1754586	1750779	99.78%	1738600	99.30%	1735701	99.14%	774.12	99.68%	99.35%	97.80%
G_3	1270486	1268634	99.85%	1257986	99.16%	1257465	99.12%	589.20	99.58%	99.17%	96.79%
P_3.1	347033	344831	99.37%	341804	99.12%	337384	97.84%	151.83	99.22%	97.92%	73.10%
P_3.2	2229305	2226881	99.89%	2210514	99.27%	2210736	99.27%	1106.57	99.66%	99.25%	96.96%
R_3	1273496	1271581	99.85%	1260829	99.15%	1260401	99.12%	585.98	99.81%	99.13%	96.63%
G_4	1176543	1174848	99.86%	1164795	99.14%	1166660	99.30%	533.51	99.65%	99.20%	96.61%
P_4.1	1209329	1207515	99.85%	1196738	99.11%	1195155	98.98%	563.18	99.58%	99.05%	95.55%
P_4.2	1611485	1609776	99.89%	1594175	99.03%	1601464	99.48%	780,90	99.58%	99.00%	96.44%
R_4	1683230	1680747	99.85%	1666277	99.14%	1663741	98.99%	797.61	99.75%	99.18%	97.39%
G_5	1887852	1885466	99.87%	1871576	99.26%	1870292	99.20%	865.72	99.84%	99.44%	98.04%
P_5.1	2541153	2538032	99.88%	2517759	99.20%	2514590	99.08%	1226.84	99.87%	99.42%	98.30%
P_5.2	1584389	1582413	99.88%	1569409	99.18%	1569898	99.21%	795.36	99.72%	99.19%	96.31%
R_5	977297	975918	99.86%	968434	99.23%	966357	99.02%	442.43	99.60%	99.02%	95.64%
G_6	1529259	1526838	99.84%	1512626	99.07%	1514121	99.17%	650.81	99.62%	99.19%	97.05%
P_6	1655540	1653328	99.87%	1639102	99.14%	1640651	99.23%	814.83	99.78%	99.25%	96.37%
G_7	1165060	1163591	99.87%	1154044	99.18%	1155029	99.26%	546.91	99.55%	98.88%	94.85%
P_7	1037344	1036074	99.88%	1027597	99.18%	1027739	99.20%	479.76	99.79%	99.24%	96.41%
G_8	1234237	1232757	99.88%	1224334	99.32%	1223610	99.26%	602.18	99.57%	98.85%	93.66%
P_8	1395362	1393608	99.87%	1383797	99.30%	1383998	99.31%	684.01	99.80%	99.33%	96.25%
G_9	1958668	1956114	99.87%	1942174	99.29%	1937388	99.04%	954.16	99.63%	99.14%	96.73%
P_9	1500042	1498180	99.88%	1486838	99.24%	1487056	99.26%	733.33	99.76%	99.21%	95.98%
G_10	1722617	1720447	99.87%	1704337	99.06%	1710391	99.42%	767.99	99.61%	99.22%	96.89%
P_10	1185363	1184138	99.90%	1172316	99.00%	1175243	99.25%	587.74	99.65%	98.76%	94.09%

To detect copy number variants (CNVs), we use Illumina GenomeStudio 2.0 and its plugin cnvPartition v3.2.1. Thresholds are set to a minimum of 100 covered probes. Intensity-only calls are excluded to increase calling precision. All remaining parameters are set to default values. In addition to automatic CNV calling, all genome-wide B allele frequency/log R ratio (BAF/LRR) plots are inspected manually.

1.7 Fluorescent *in situ* hybridization

All flurescent *in situ* hybridization (FISH) analyses were performed in the genetic reference institutions of the NHL-BFM study group.

1.8 Categorizing clonal evolution

To categorize clonal evolution at two time points, we propose the following 3 main categories of clonal evolution: independent evolution, parallel dependent evolution and non-parallel dependent evolution, i.e. linear evolution (see table S1).

Further subcategories can be defined for the main categories "non-parallel dependent evolution" and "independent evolution". If two (sub)clones co-exist at both time points, with no (sub)clone presenting itself as the dominant one at any time, we refer to this subcategory as "continued co-existence". Contrary, one (sub)clone may be dominant at both time points. This subcategory is referred to as "continued dominance".

If (sub)clone 1 is dominant at time point 1, but loses its dominance as both (sub)clones co-exist at time point 2, we refer to this subcategory as "gained co-existence'. If (sub)clone 2 is dominant at time point 2, whereas it has not been dominant at time point 1, this is a "gained dominance". It should be noted that both dominance of (sub)clone 1 and co-existence of (sub)clones 1 and 2 at time point 1 could have occurred.

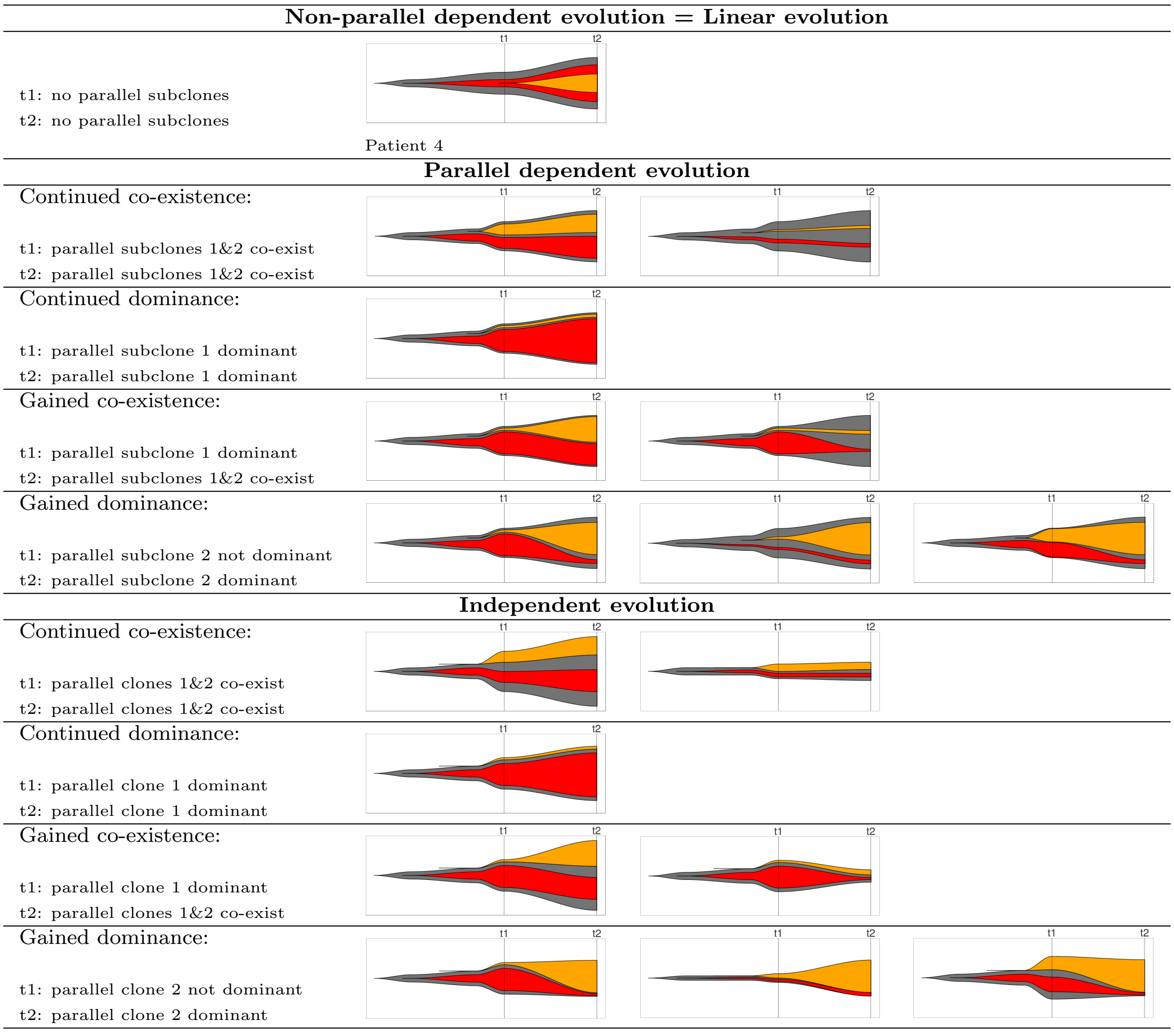

Figure S1: Categorizing clonal evolution. Categories for clonal evolution at two time points. Three main categories can be distinguished: independent evolution, parallel dependent evolution and non-parallel dependent evolution, i.e. linear evolution.

1.9 Reconstructing clonal evolution

To reconstruct clonal evolution of all patients, we followed the approach applied by da Silva-Coelho *et al.* 2017 [1]. Clonal evolution patterns are constructed manually, based on mutation calling results.

We consider all somatic SNVs and indels detected in WES data. For these calls, we evaluate VAFs based on WES data and – if available – also based on targeted data. Additionally, we consider CNV calling information from SNP array analysis. Based on reported beta allele frequencies (BAFs) and – whenever possible – frequency of variant calls in WES data (comparing germline vs tumor) we estimate the fraction of cells affected by CNV (see section 1.9.1, for details on the estimated cell fractions see Supplementary Data 2 A).

To reconstruct clonal evolution, we correct VAFs of SNVs and indels for CNV information. Subsequently, mutations are clustered considering all available time points. Finally, clonal evolution is derived.

It has to be noted that we can only analyze 1 time point in case of non-relapse samples, and 2 time points in case of relapse samples (in comparison to germline information, which we use as baseline values). For non-relapse samples, it is not possible to analyze the change in VAF over time. A draft of clonal evolution can only be reconstructed by clustering mutations

at primary diagnosis.

To optimize visualization of clonal evolution – including up to 17 subclones – by the R package "fishplot" version 0.5 [20], we estimate development of the clones over time (see section 1.9.2 and the effect of therapy (see section 1.9.3).

1.9.1 Estimating cell fractions for CNVs

The relation between VAF of a polymorphism and the fraction of cells affected by a (simple) deletions or duplications is visualized in Figure S2.

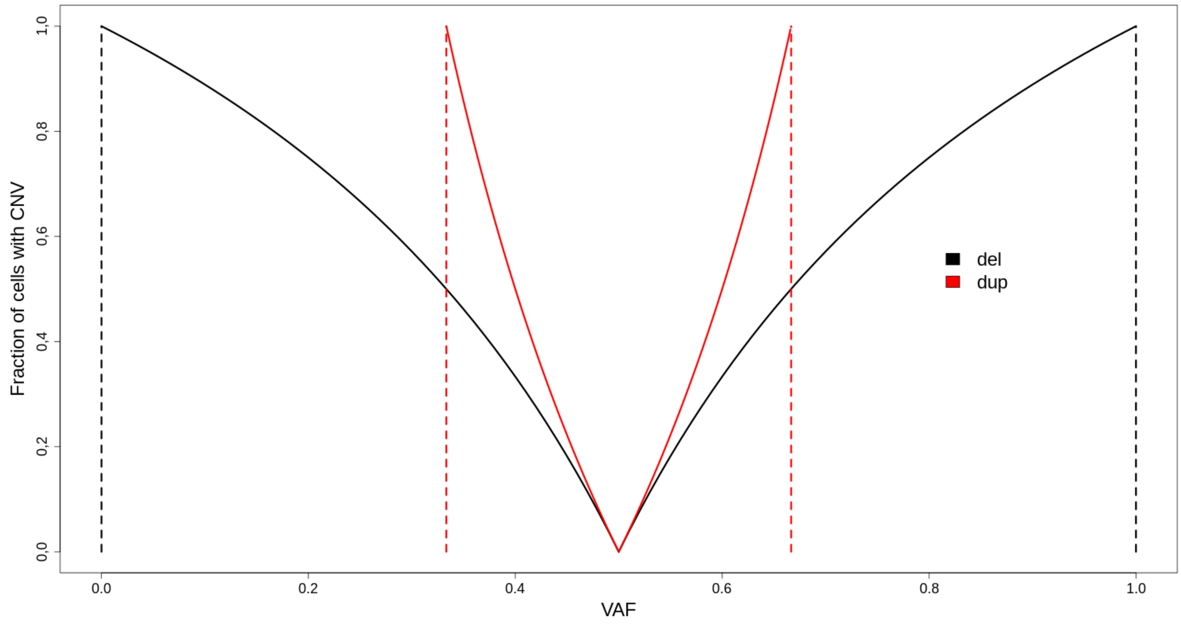

Figure S2: Relation between VAF of a polymorphism and fraction of cells affected by CNV. Copy number variations lead to a change in VAF of heterozygous polymorphisms. If the fraction of cells affected by CNV is unknown, the observed change in VAF can be used for estimation (deletions: black; duplications: red).

For SNP array data, the BAFs as reported by GenomeStudio are analyzed. For every region affected by CNV, we evaluate the BAFs of all SNPs located within this region. The mean fraction of cells as well as standard deviation are calculated and reported in Supplementary Data 2 A.

Additionally, we consider WES data. To identify likely heterozygous polymorphisms, we perform variant calling on the germline samples using VarDict (minimum depth=30, minimum number of alternate reads=10, minimum VAF=0.2). A variant is considered likely heterozygous if the 95% confidence interval for VAF covers 0.5. For all these calls, we determine VAFs in the matching tumor samples. Similar to the analysis of SNP array data, we calculate the fraction of cells affected by CNV based on VAF. To account for the fact that some regions may be characterized by a systematically higher or lower VAF, we compare cell rates to those calculated on the basis of matching germline samples. The mean fraction of cells, standard deviation and the number of evaluated mutations are reported in Supplementary Data 2 A.

In case of some regions, no or few mutations are detected in WES data. For regions with <10 mutations, we symmetrically increase the investigated region until ≥ 10 mutations can be analyzed. The mean fraction of cells, standard deviation, number of evaluated mutations and genomic coordinates of the evaluated regions are reported in table Supplementary Data 2 A.

1.9.2 Estimating development of clones over time

When considering clonal evolution, it is sensible to assume that the different mutations characterizing the clones are not acquired within hours. Instead, it appears more likely that some time has passed while the clones develop and additional mutations are acquired. Clonal evolution plots generated with “fishplot” only partly show this development: While subclones emerging before time point 1 show the expected development, time between emergence of two subclones decreases continuously. This makes it hard to identify the later subclones in the plots. At a second time point, successive emergence of subclones is no longer visible at all. Probably, time between the subclones has decreased further (see results in section 2.3.3).

It should be noted that “fishplot” supports, by default, only 10 subclones. However, clonal evolution of patients 1 and 5 feature 17 subclones. It is reasonable to assume that the default “fishplot”-assumptions – successive development of subclones with continuously decreasing time between two subclones – have to be adapted to fit our study.

To illustrate our approach, we consider an example with only 4 mutations. Each mutation characterizes an individual clone. The mutations are acquired successively, i.e. we are dealing with linear evolution. The reconstructed clonal evolution and the percentage of cells with the mutations (maximum 100%), measured at 2 time points, are visualized in Figure S3.

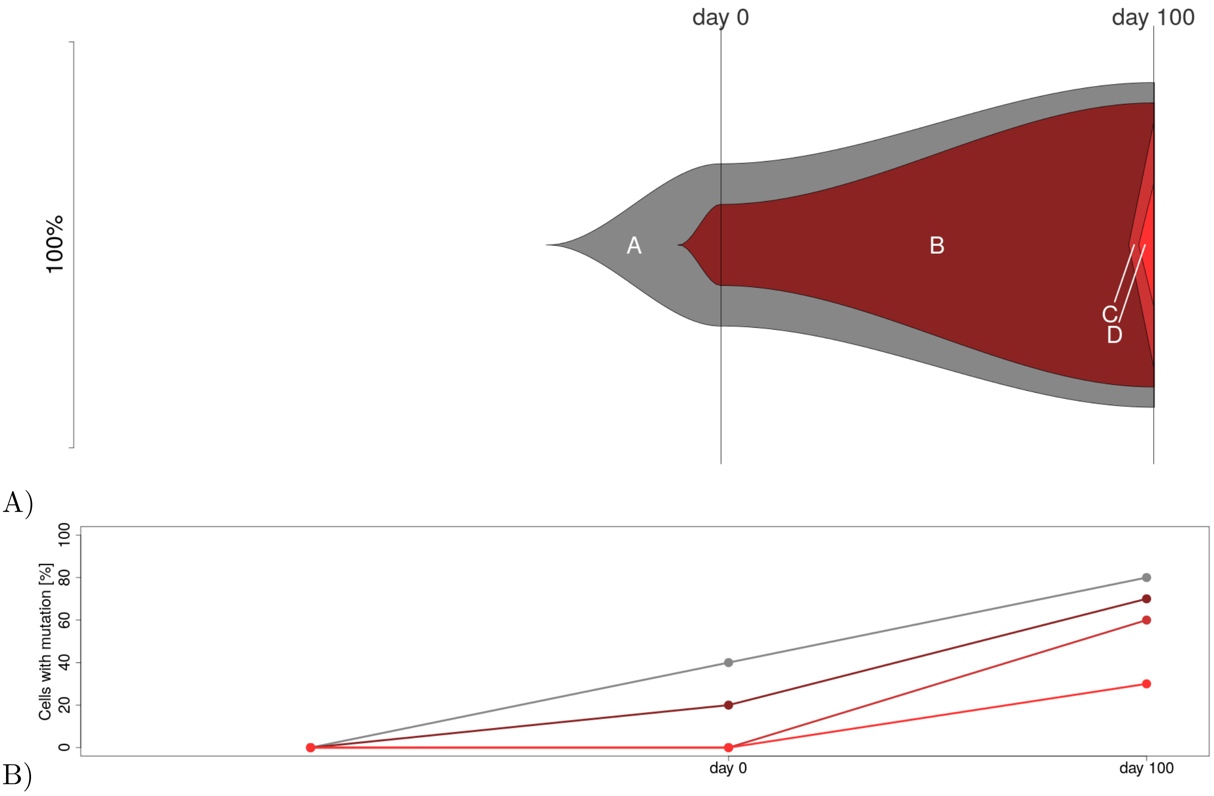

Figure S3: Exemplary clonal evolution without estimated time points. Example for linear clonal evolution, including 1 clone and 3 subclones with measurements at 2 time points. Every (sub)clone is characterized by one mutation each. A) Clonal evolution. B) Percentage of cells with mutations.

It can be observed that the time passing between emergence of different subclones is – according to Figure S3A – different. Already, identification of subclones C and D appears difficult.

To improve visualization of clonal evolution with high as well as low number of subclones, we assume equidistant development of subclones. To realize this approach with “fishplot”, we

have to estimate development of our subclones over time and add estimated time points to our measured ones.

For reasons of simplicity, we assume that the time span between our two measured time points “day 0” and “day 100” is as long as the time span between “day 0” and the beginning of our clonal evolution, i.e. acquisition of mutation A in our example. The reconstructed clonal evolution and the percentage of cells with the mutations, measured at 2 time points and estimated at additional time points, are visualized in Figure S4.

Two mutations – A and B – are acquired between the baseline value and the first measurement at day 0. At the intermediate time point – at day -50* –, we assume that only mutation A is present. Furthermore, we assume that the percentage of cells with mutation A at day -50* is the same as cells only featuring mutation A at day 0, i.e. 20%.

Following this principle, we estimate time point day 50*: mutation C is already present, while mutation D is not. Percentage of cells with mutation C at day 50* is the same as cells only featuring mutation C at day 100, i.e. 30%.

The plot resulting from these assumptions can be found in Figure S4A. Considering our last mutation D, visualization is still not optimal. Furthermore, the increase in cells for the different subclones appears quite abrupt, e.g. in case of subclone C.

To generate plots with a smoother development of clonal evolution, we add the proportion of 0.1% – indicating that the clone has just emerged, but was still below detection thresholds – for each clone to the previous time point: At day -100*, clone A is present in 0.1% of the cells. At day -50*, subclone B is present in 0.1% of the cells. At day 0, subclone C is present in 0.1% of the cells. At day 50*, subclone D is present in 0.1% of the cells. The resulting plot can be found in Figure S4B.

1.9.3 Estimating effect of therapy

Despite providing an already optimized visualization of clonal evolution, the plot in Figure S4B is still characterized by two possible shortcomings: 1) We know that therapy was applied between our 2 measured time points. However, no effect of therapy on clonal evolution can be observed in the plot. 2) Comparing day 50* to day 100, a decrease in the percentage of cells only featuring mutations A and B can be observed.

It is of course possible that therapy has, e.g. due to resistance, a reduced effect on clonal evolution. Furthermore, it is possible that percentages of clone A and subclone B decrease due to being pushed away by subclones C and D. However, in our study, we know from additional examinations that therapy did – at least initially – always have the desired effect on clonal evolution of patients 1 to 10. Therefore, in our example, we assume that therapy has an effect on clonal evolution as well, and that the effect becomes apparent by the decreasing presence of subclones A and B at day 100.

Reconstructed clonal evolution and the percentage of cells with the mutations, considering an estimated effect of therapy, are visualized in Figure S5.

Comparing our 2 measured time points, day 0 and day 100, it can be observed that the percentage of cells only carrying mutation A decreases from 20% to 10%. Furthermore, the percentage of cells carrying mutations A and B decreases from 20% to 10%. As we assume this decrease to be caused by therapy, we add an additional time point with 10% of the cells featuring mutations A and B, and 10% of the cells featuring only mutation A. Sticking to our principle of equidistant development, we shift our previous time point day 50* to day 67* and generate a new estimated time point “day 33*”, visualizing the effect of therapy.

This 2-step procedure of estimating additional time points – optimizing visualization of clonal evolution and estimating the effect of therapy – is applied on all patients to generate the final clonal evolution plots shown in Figure 2 A-E.

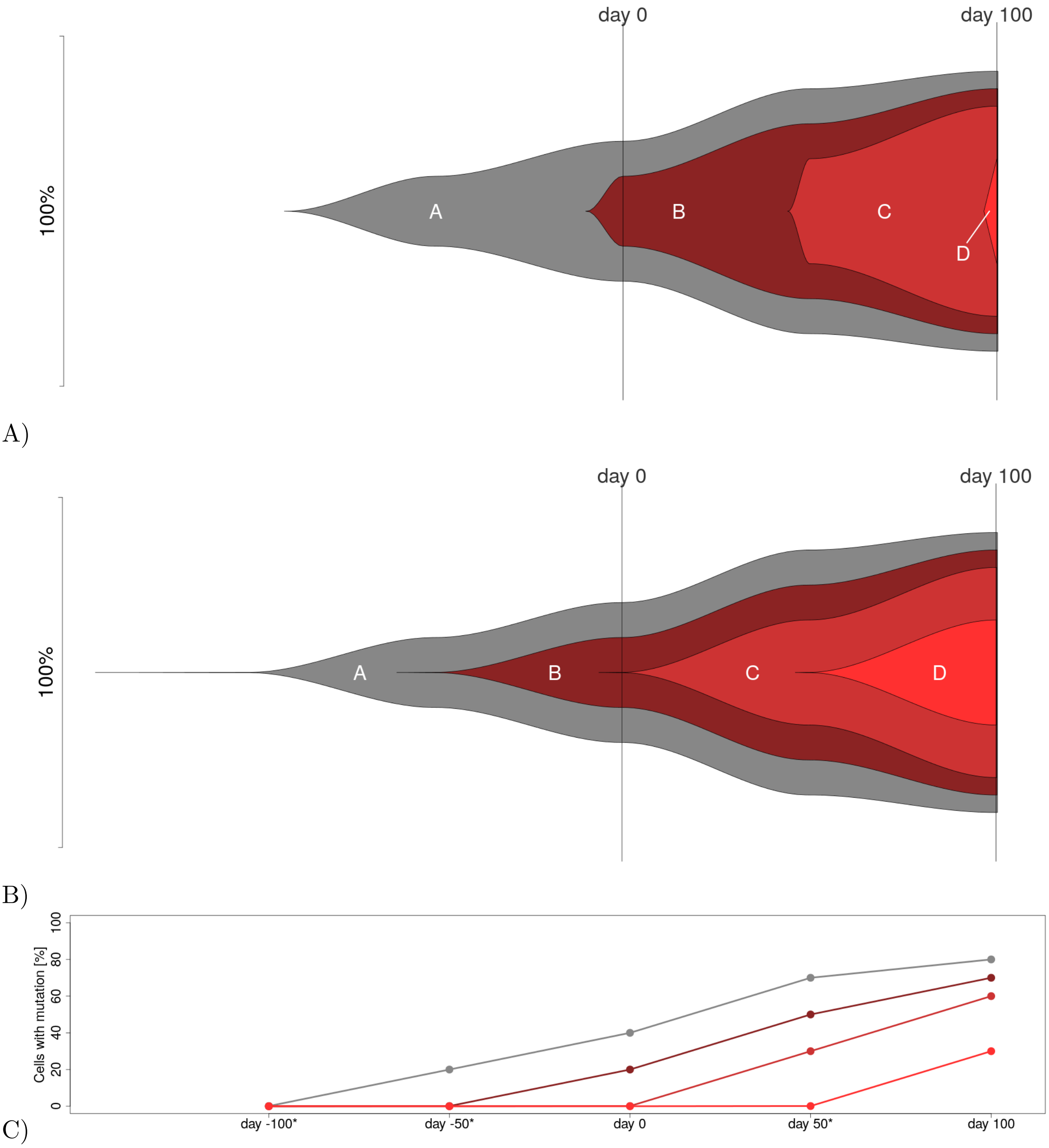

Figure S4: Exemplary clonal evolution with estimated time points. Example for linear clonal evolution, including 1 clone and 3 subclones with measurements at 2 time points and estimated cells with mutations at additional time points. Every (sub)clone is characterized by one mutation each. A) Clonal evolution with 2 estimated time points. B) Clonal evolution with 3 estimated time points. C) Percentage of cells with mutations, estimated time points are marked with an asterisk.

1.9.4 Automatic approaches

In addition to manual reconstruction of clonal evolution, we consider common tools for automatic reconstruction: To identify clones via clustering we apply PyClone [21] and SciClone [22]. For subsequent clonal ordering and visualization, we consider ClonEvol [23].

PyClone is applied using the function `run_analysis_pipeline` with default parameters.

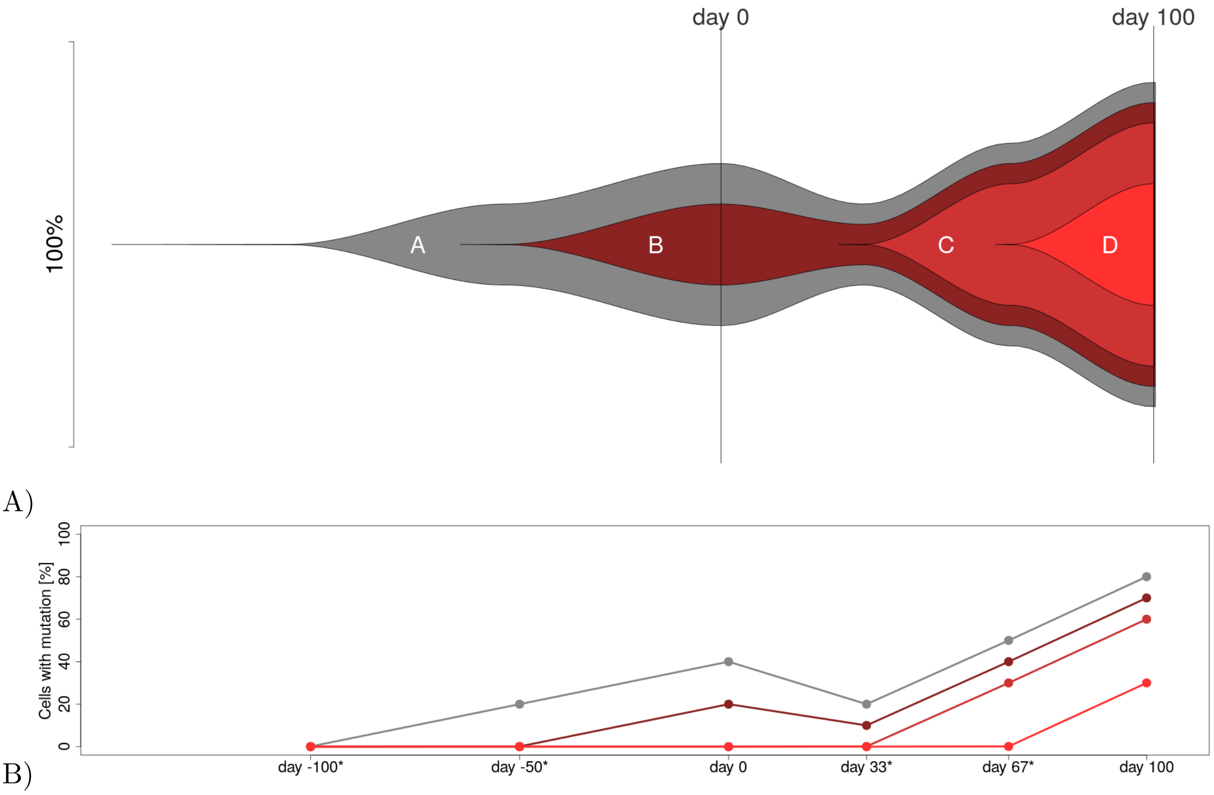

Figure S5: Exemplary clonal evolution with estimated time points and estimated therapy effect. Example for linear clonal evolution, including 1 clone and 3 subclones with measurements at 2 time points and estimated cells with mutations at 4 additional time points. Every (sub)clone is characterized by one mutation each. A) Clonal evolution. B) Percentage of cells with mutations, estimated time points are marked with an asterisk.

Information on SNVs and indels is provided as `in_files`. Information on copy number variants could be considered in case they were overlapping detected mutations. Parameters `minor_cn` and `major_cn` were adjusted. Additional CNVs, not overlapping any detected mutations, could not be taken into account. Furthermore, translocations (here: C-MYC rearrangement for all patients) could not be taken into account. It should be noted that the developer of PyClone recommends its usage only for reconstructing clonal evolution on the basis of SNV information.

SciClone is used, following the standard workflow for clustering two samples (relapse patients). For every sample, information on SNVs and indels (including the number of reference reads as `ref_reads`, the number of reads with the variant as `var_reads` and the variant allele frequency as `vaf`) as well as copy number variants (including the position and copy number value) are analyzed. Translocations (here: C-MYC rearrangement for all patients) could not be taken into account. As proposed by the standard workflow, regions with detected loss of heterozygosity are defined as `regionsToExclude`.

ClonEvol is applied on the output generated by PyClone and SciClone for all relapse patients. We use ClonEvol, following the workflow described in "A tutorial on clonal ordering and visualization using ClonEvol".

2 Supplementary Results

2.1 SNV and indel calling

High-coverage WES analysis reveals in total 481 somatic SNVs and indels. Distribution of the SNVs and indels over all patients (5 primary vs relapse, 5 primary) is visualized in Figure S6.

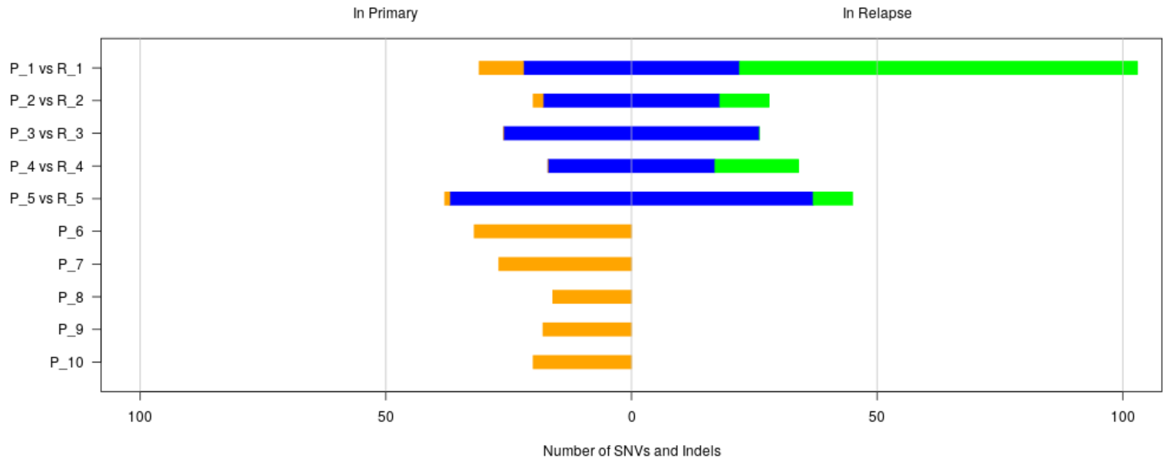

Figure S6: Summarized SNV and indel calls. Distribution of SNVs and indels over all patients (orange: primary specific; green: relapse specific; blue: shared mutations).

It can be observed that all relapse patients are characterized by a high level of shared mutations. The only exception – due to a considerably higher mutation rate in relapse – is patient 1. This patient features 103 somatic mutations in relapse compared to only 31 mutations in primary sample. Ninety-one out of 103 mutations are relapse-specific.

A graphical overview of the most frequently mutated genes in our cohort can be found in Figure S7.

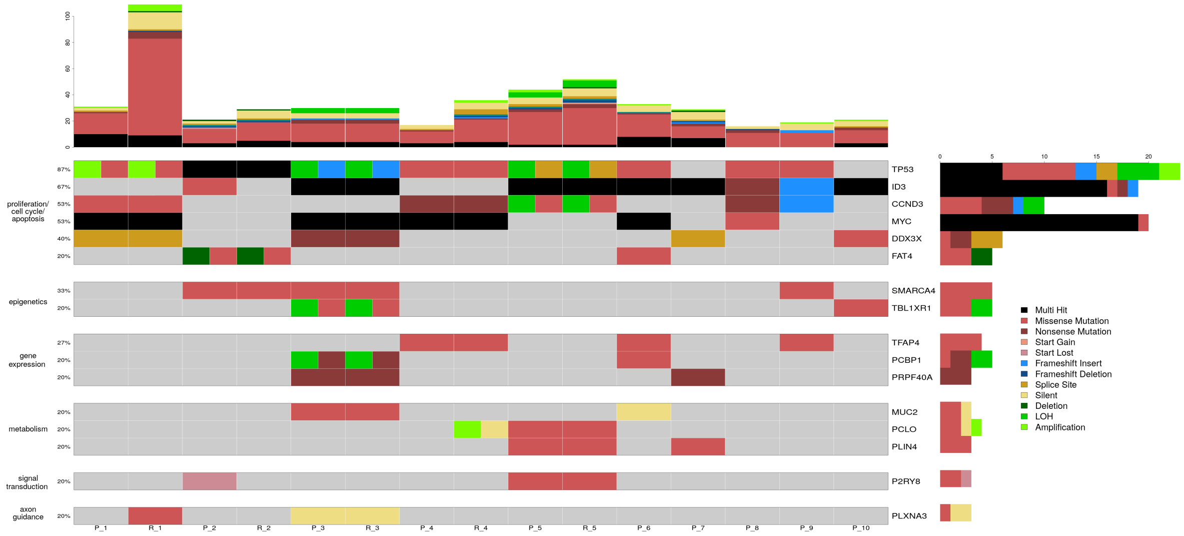

Figure S7: Oncoplot of the most frequently mutated genes. Graphical overview of the most frequently mutated genes, including SNVs, indels and CNVs. Genes affected by mutations are clustered according to affected pathway.

It can be observed that most genes affected by mutations belong to the pathway “proliferation/cell cycle/apoptosis”.

To investigate if the identified pathway “proliferation/cell cycle/apoptosis” is significantly enriched, we performed gene enrichment analysis using DAVID [24, 25]. Using “Functional Annotation”, we analyzed the list of genes carrying somatic mutations as detected by WES, excluding all silent mutations (Supplementary Data 1 A). “OFFICIAL_GENE_SYMBOL” was selected as identifier, “Gene list” for list type. As species, “Homo sapiens” was selected (235 out of 236 genes identified). The results for KEGG are summed up in table S6.

Table S6: Gene enrichment analysis. KEGG pathways reported from gene enrichment analysis with DAVID. No significant results can be observed when considering the adjusted p value.

KEGG Pathway	Count	Genes	P value	Adjusted p value (Benjamini)
Cell cycle	8	*CCNB3, CDC45, CCND3, RBL2, TP53, RB1, MYC, MCM5*	0.00	0.29
Chronic myeloid leukemia	5	B*CR, TP53, SHC1, RB1, MYC*	0.02	0.81
Pathways in cancer	12	*LAMA2, GNA13, DCC, LAMA1, BCR, GNAQ, NTRK1, ARNT2, TP53, FOXO1, RB1, MYC*	0.03	0.77
Small cell lung cancer	5	*LAMA2, LAMA1, TP53, RB1, MYC*	0.03	0.76
Wnt signaling pathway	6	*TBL1XR1, CHD8, CCND3, TP53, MYC, TBL1Y*	0.05	0.81
Cell adhesion molecules (CAMs)	6	*NCAM1, ALCAM, ITGAL, ITGA8, MADCAM1, CNTNAP1*	0.06	0.79
Thyroid cancer	3	*NTRK1, TP53, MYC*	0.07	0.80
Viral carcinogenesis	7	*CCND3, HIST1H4A, DDX3X, RBL2, TP53, HDAC10, RB1*	0.08	0.81
Transcriptional misregulation in cancer	6	*NTRK1, ARNT2, TP53, FOXO1, MYC, DDIT3*	0.10	0.84

Despite our observations in Figure S7, no pathway was identified to be significantly enriched. However, the KEGG pathway with the lowest adjusted p value (cell cycle) matches our pathway with the highest number of genes affected by mutations (proliferation/cell cycle/apoptosis).

To determine likely driver genes, we analyze our variant calling results with MutSigCV_1.41. An overview of the output, including the top-10 significant genes, is provided in table S7.

Table S7: MutSigCV output. MutSigCV output-file sig_genes.txt including the top-10 significant genes (N_nonsilent, number of covered sequenced bases containing non-silent mutations; N_silent, number of covered sequenced bases containing silent mutations; N_noncoding, number of covered sequenced bases containing noncoding mutations; n_nonsilent, number of non-silent mutations; n_silent, number of silent mutations; n_noncoding, number of noncoding mutations; nnei, number of neighboring genes; x, number of mutated bases in neighboring genes; X total number of bases related to neighboring genes; p, p-value; q, q-value, i.e. adjusted p-value). q values indicate that 4 genes are significant with $\alpha < 0.05$: *CCND3*, *DDX3X*, *ID3* and *TP53*.

gene	N_nonsilent	N_silent	N_noncoding	n_nonsilent	n_silent	n_noncoding	nnei	x	X	p	q
TP53	46335	12795	0	17	0	0	50	0	777375	2.10e-14	**3.96e-10**
CCND3	30525	9750	0	8	0	0	50	0	769155	9.33e-10	**8.80e-06**
ID3	12495	4065	0	18	1	0	5	1	54300	1.06e-07	**6.67e-04**
DDX3X	73620	18765	0	6	0	0	50	0	887865	2.36e-07	**1.41e-03**
IGLL5	22305	7080	0	4	0	0	50	0	669405	6.19e-05	2.33e-01
RAB11B	23775	6600	0	2	0	0	50	0	862800	5.34e-04	1
PCBP1	36660	11535	0	3	0	0	50	0	672915	1.36e-03	1
*PRPF40*A	105300	24975	0	3	0	0	50	0	884535	1.90e-03	1
GNA13	40530	11040	0	3	0	0	50	0	703965	2.66e-03	1
ADAD1	63450	16515	0	2	0	0	50	0	767160	5.13e-03	1

Evaluating the adjusted p value – the q value – 4 genes are significant with $\alpha < 0.05$: *CCND3*, *DDX3X*, *ID3* and *TP53*. Figure S7 shows that all of these genes belong to the same, mostly affected pathway “proliferation/cell cycle/apoptosis”.

Detailed analysis of mutations in *CCND3*, *DDX3X*, *ID3* and *TP53* – considering relapse and non-relapse patients separately – is visualized in Figure S8. It can be observed that all

driver genes are affected differently by mutations in relapse vs non-relapse Burkitt lymphoma.

Both *CCND3* and *DDX3X* do not feature a single mutation, which is shared by relapse and non-relapse patients. *ID3* carries known hotspot mutations for BL [26]: in our cohort, 2 of which are shared in relapse and non-relapse patients (Q81* and P56S). One is detected in non-relapse patients only (L64F). Moreover, 6 additional mutations in *ID3* are identified in non-relapse patients and 2 only in relapse patients. Similarly, the common hotspot mutation R248Q in *TP53* is detected in relapse and non-relapse patients [27]. Five additional *TP53* mutations are exclusively present in patients with relapse.

It should be noted that all non-relapse patients carry additional point mutations in potentially good prognostic genes – none of which was detected in relapsing patients: *SYNCRIP* (nonsense mutation in patient 6; depletion may delay lymphomagenesis), *SRSF7* (missense and nonsense mutation in patient 7; silencing may decrease proliferation), *DDIT3* (missense mutation in patient 8; induces p53-independent apoptosis), *SASH1* (missense mutation in patient 9; positive correlation with a better post-operative survival in glioma patients), *ZMIZ1* (missense mutation in patient 10; can activate *TP53*) [28, 29, 30, 31, 32].

As relapse and non-relapse patients are characterized by different mutation profiles, patients with relapse might be characterized by mutations that are more damaging. Furthermore, mutations found in relapse and non-relapse patients could, in general, be less damaging. To investigate these hypotheses, we performed *in silico* evaluation of all mutations. We used Provean (http://provean.jcvi.org) to predict the effect of all somatic SNVs and indels detected in our samples. The results with respect to prediction score are visualized in Figure S9 (precise values are provided in Supplementary Data 1 A).

Histograms in Figure S9 show that – according to Provean prediction score – patients with relapse are not in general characterized by more mutations with lower prediction scores.

Considering TP53 mutations in particular, we detect 10 mutations. For one frameshift (patient 3) and one splice-site mutation (patient 5), no Provean prediction is available. For the remaining 8 missense mutations, Provean prediction is 'damaging' for all but 2 mutations. These two exceptions are mutations we detect in patient 2. Detailed analysis of this variants shows that they are located within the coding region of TP53 according to only 4 out of 15 transcripts (different from R248Q or C289R, which are located in the coding region according to all transcripts and which have a damaging prediction). Assuming that this might bias the Provean prediction, we performed additional analyses using PolyPhen2 [33]. By manually adding the FASTA sequences, the website allows for an isoform-specific prediction. For the first variant, changing Phenylalanine to Leucine, PolyPhen2 predicts a 'possibly damaging' effect (score 0.954) for all 4 isoforms. For the second variant, changing Leucine to Serine, PolyPhen2 predicts a 'benign' effect (score 0) for all 4 isoforms.

Thus, although we cannot observe any differences between relapse and non-relapse samples based on *in silico* analyses, the algorithm used for prediction seems to have a considerable influence on the predicted effect. It is possible that a true difference between the two subgroups is not observed as the true effect of each mutation cannot be estimated validly.

2.2 CNV calling

SNP array analysis reveals in total 93 CNVs. Distribution of the CNVs over all patients (5 primary vs relapse, 5 primary) is visualized in Figure S10.

It can be observed that all non-relapse patients are characterized by a low number of CNVs (average: 1.6 CNVs/sample). No non-relapse patient is characterized by a non-complex karyotype, i.e. ≤ 3 CNVs per patient [34]. Relapse patients feature a higher number of CNVs (average primary: 7.4 CNVs/sample; average relapse: 9.4 CNVs/sample), including a high level of shared CNVs in all patients except for patient 4. Altogether, only 4 CNVs are primary-specific and 14 CNVs are relapse-specific. Thirty-three CNVs are shared by primary and relapse.

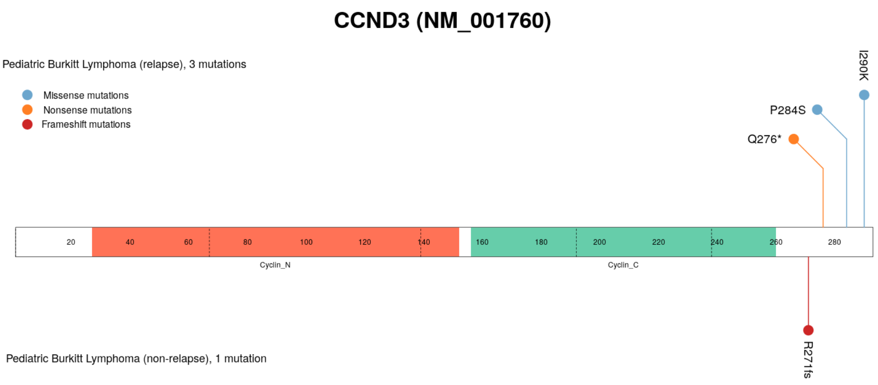

A)

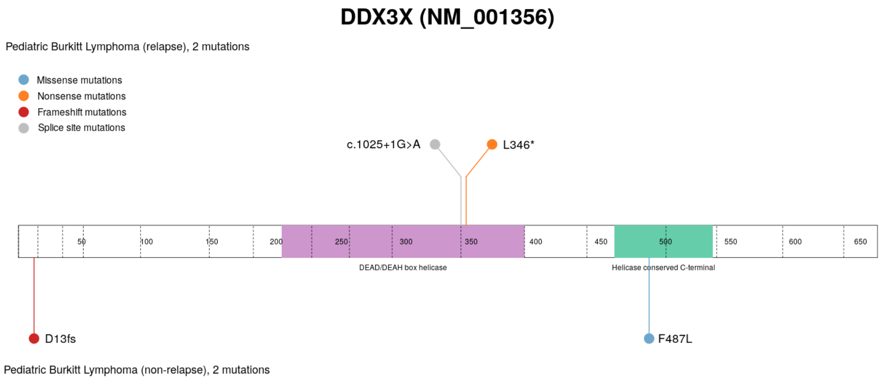

B)

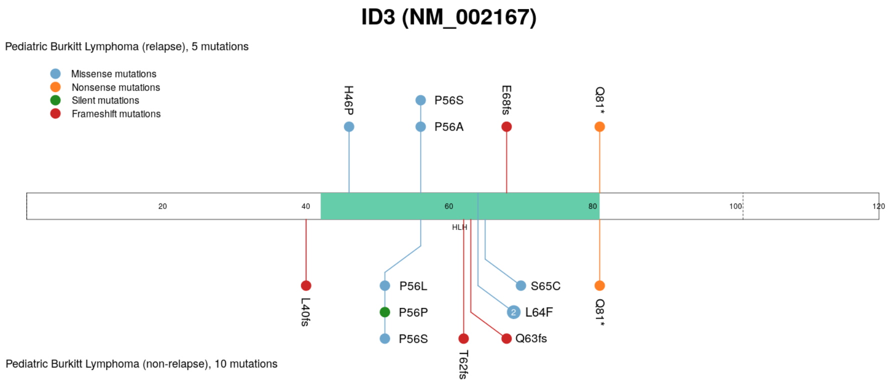

C)

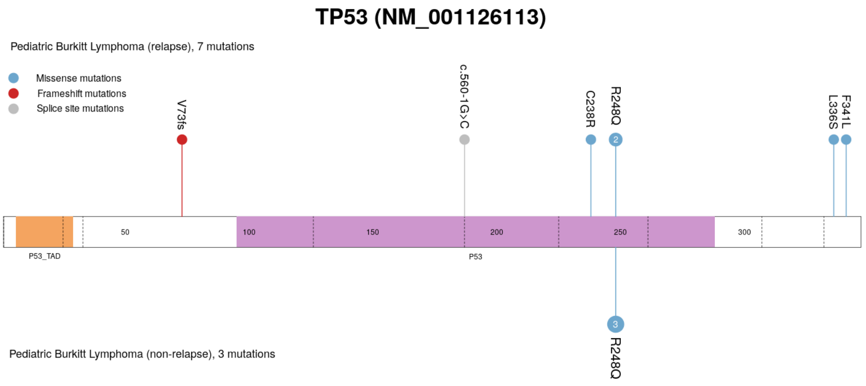

D)

Figure S8: Lollipop plots for significantly mutated genes. Mutations detected in A) *CCND3*, B) *DDX3X*, C) *ID3*, D) *TP53* in relapse and non-relapse patients. All genes have been identified as significant driver genes.

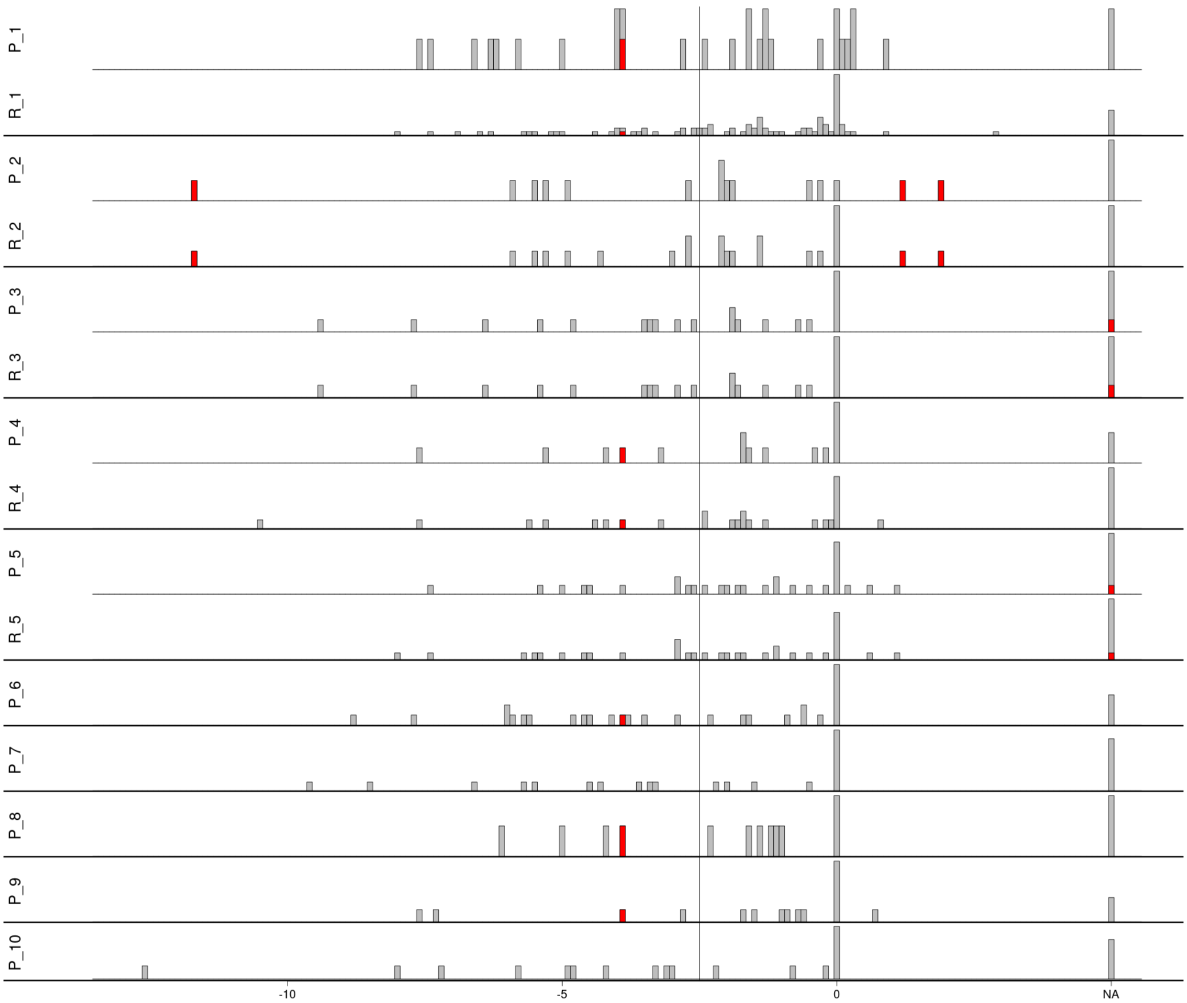

Figure S9: ***In silico*** **evaluation of mutations.** Provean prediction scores for somatic mutations detected in primary and relapse samples (default cutoff for deleterious < -2.5). Prediction scores for mutations in TP53 are marked in red. Missing prediction scores (NA) result from frameshift mutations and splice site mutations.

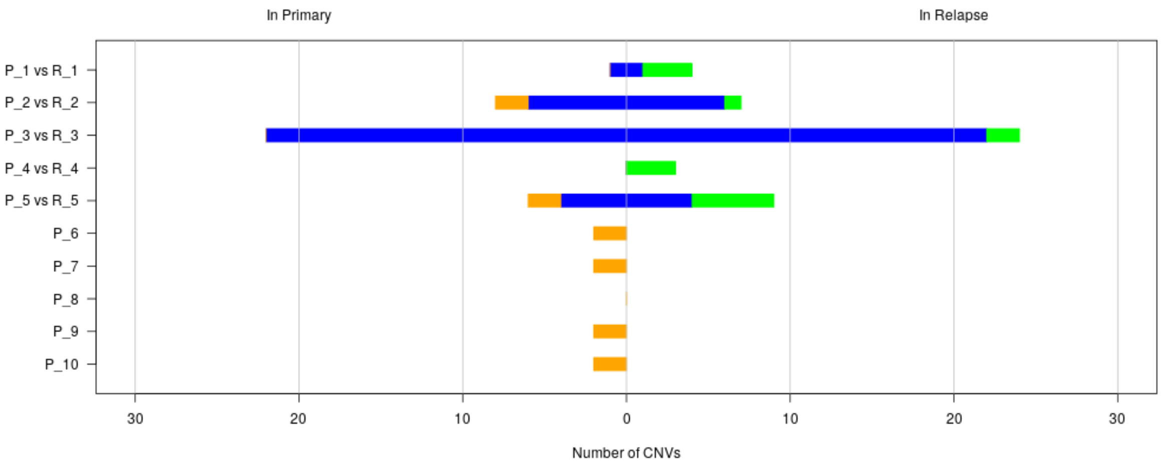

Figure S10: Summarized CNV calls. Distribution of CNVs over all patients (orange: primary specific; green: relapse specific; blue: shared mutations).

Three out of 5 patients with relapse already show complex karyotype at the time of primary diagnosis, and 5 out of 5 at the point of relapse.

A detailed visualization of all CNVs – deletions, loss of heterozygosity (LOH) and duplications – detected in every sample is provided in Figure S11.

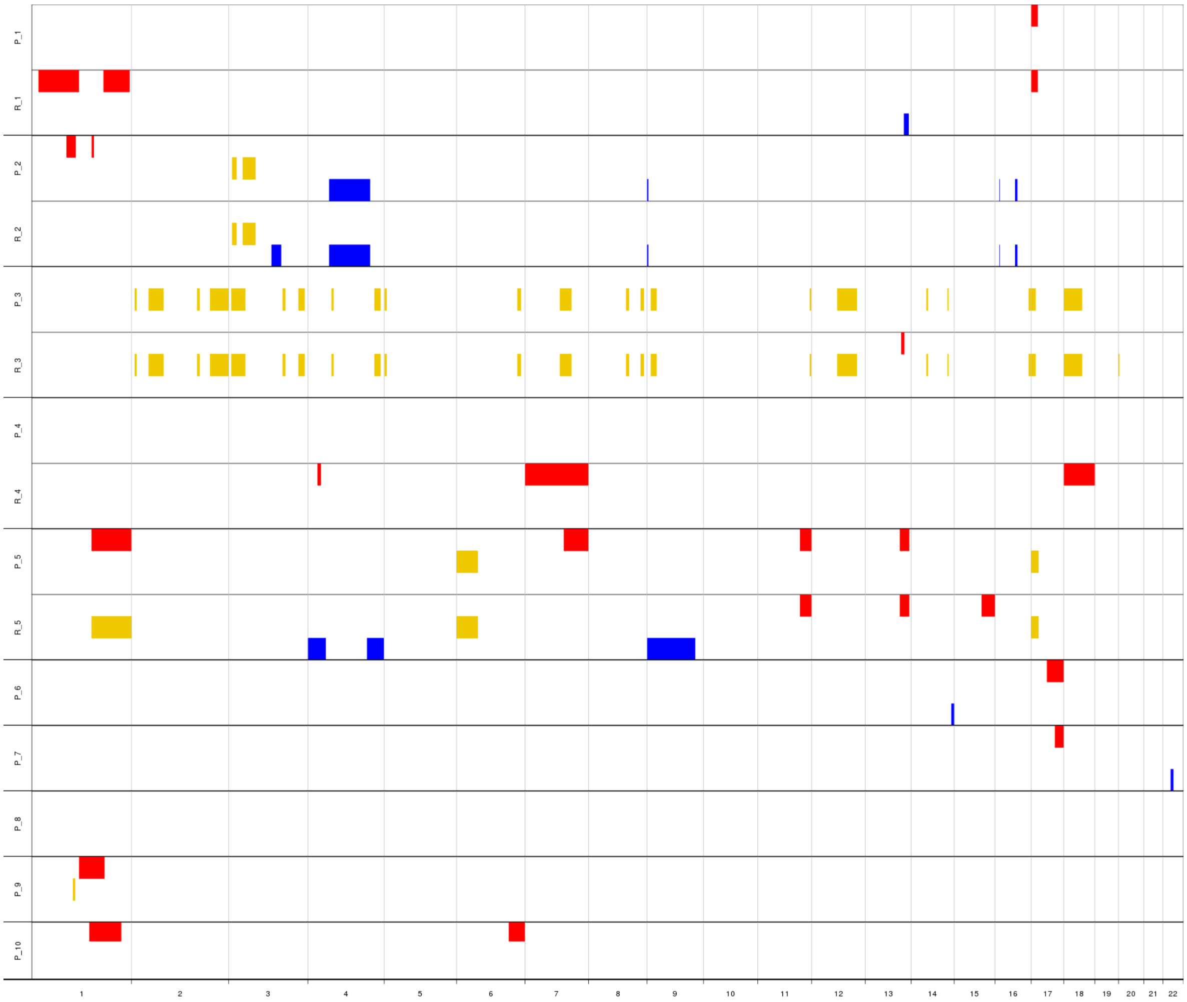

Figure S11: Detailed CNV calls. CNVs detected for every patient (blue: deletion; yellow: LOH; red: duplication).

The high level of shared mutations in 4 out of 5 relapse patients, already visible in Figure S10, can also be observed in Figure S11. However, comparing different patients, differences regarding chromosome/position and copy number can be observed. In patient 1, one deletion is shared. Patient 2 features shared LOHs and deletions comparing primary to relapse. For patient 3, only LOHs are shared. Patient 5 features shared duplications and LOHs.

Evaluating CNV positions, a specific genomic region affected by CNVs more often – dependent or independent of relapse – cannot be identified.

2.2.1 CNV calls on chromosome 17

Detailed evaluation of SNP array data reveals an interesting case for chromosome 17. Plots visualizing BAF and log R ratio (LRR) for patients 4 and 5 (primary and relapse) as well as patients 6 to 10 (primary) can be found in Figure S12. The position of *TP53* is marked by a red line.

It can clearly be observed that patient 5 features a CNV at the beginning of chromosome 17 (compare to Figure S11). Similarly, the called CNVs for patients 6 and 7, located towards

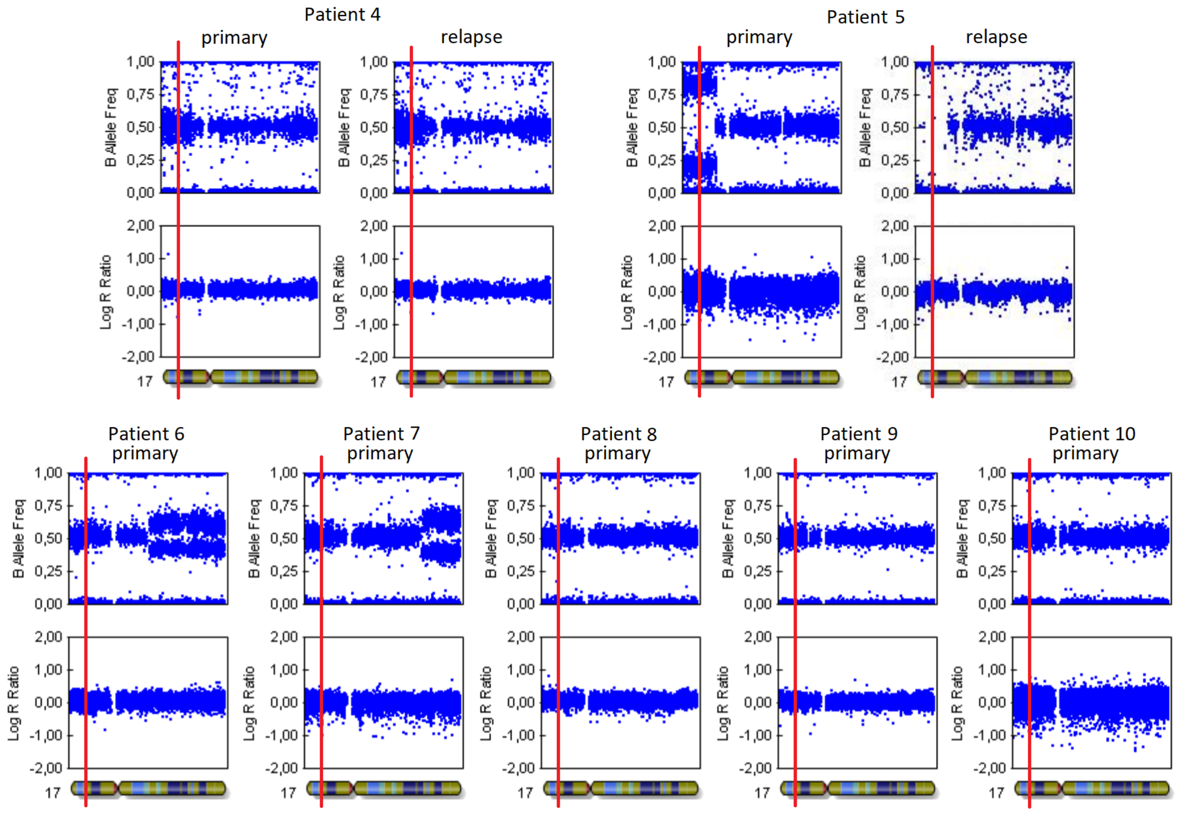

Figure S12: CNVs on chromosome 17. SNP array data showing chromosome 17 (plots visualizing B allele frequency and log R ratio, generated by Illumina GenomeStudio 2.0) for patients 4 and 5 (primary and relapse) as well as patients 6 to 10 (primary). The red lines mark the position of *TP53*.

the end of chromosome 17, (Figure S11) can also be observed in Figure S12.

When considering non-relapse patients 8 to 10, data does not indicate presence of any CNV on chromosome 17. The only variation in BAF and/or LRR that can be observed is likely to be due to background noise.

Regarding patient 4, two "bubbles" can be observed in the BAF plots: towards the beginning and the end of chromosome 17. These "bubbles" are present in both samples – primary and relapse. Comparing data to patient 5, who features a CNV at the beginning of chromosome 17 in a high fraction of cells, and patients 6 and 7, who feature CNVs at the end of chromosome 17 in a lower fraction of cells, these two "bubbles" are likely to indicate two CNVs, present in a fraction of cells that is below detection thresholds.

Thus, detailed evaluation of SNP array data indicates that *TP53* is not expected to be affected by a CNV in case of any non-relapse patient. However, it is affected in case of patient 5 (high frequency of cells) and, most likely, also in case of patient 4 (low frequency of cells).

2.3 Clonal evolution

2.3.1 Applied categorization

To systematically categorize clonal evolution, comparing two time points, we propose 3 main categories and 4 different subcategories. In table S8, the main categories and subcategories are summarized together with examples based on our data as well as patients with myelodysplastic syndrome (MDS) studied by da Silva-Coelho *et al.* 2017 [1] (UPNs).

Linear evolution is a frequent type of clonal evolution that can be observed in both, our

Table S8: Applied categorization for clonal evolution. Main categories and subcategories for clonal evolution at two time points. Information on our samples with relapse (Patients 1-5) and samples analyzed by da Silva-Coelho *et al.* 2017 [1] (UPNs) were added to the matching main- and subcategories.

Main category	Subcategory	Example
Linear evolution	/	Patient 4<br>UPN01, UPN06, UPN11
Parallel dependent evolution	Gained dominance	Patients 1, 2, 5<br>UPN03, UPN05 t2→t3, t4→t5
	Continued dominance	/
	Gained co-existence	Patient 3<br>UPN05 t1→t2, t3→t4
	Continued co-existence	/
Independent evolution	Gained dominance	UPN08 t2→t3
	Continued dominance	UPN08 t1→t2, t3→t12
	Gained co-existence	/
	Continued co-existence	/

study (patient 4) as well as in the study by da Silva-Coelho *et al.* 2017 (UPN01, UPN06 and UPN11).

For all the other relapsing patients in our study we observe parallel dependent evolution. Clonal evolution of patients 1, 2 and 5 can be categorized as gained dominance. The same subcategory of clonal evolution can also be observed for UPN03 and UPN05 (comparing time point 2 to 3, or time point 4 to 5). A different subcategory of parallel dependent evolution – gained co-existence – can be observed for patient 3 and UPN05 (comparing time point 1 to 2, or time point 3 to 4). Comparing Figure 2 A, B and E to Figure 2 C, essential differences between these two subcategories of clonal evolution can be observed.

Independent evolution cannot be observed in our study. However, in the study by da Silva-Coelho *et al.* 2017, examples for two subcategories can be found: UPN08, comparing time point 2 to 3, is characterized by gained dominance. Comparing time point 1 to 2, or time point 3 to all successive time points for this patient, continued dominance can be observed.

When categorizing clonal evolution, we aimed at defining a systematic nomenclature that does not only serve our 5 relapse samples, but that can in general be applied when comparing two time points in the context of clonal evolution. As an example, we considered the study by da Silva-Coelho *et al.* 2017. As the results in table S8 show, our proposed categorization can successfully be applied to all samples and time points with up to 2 parallel subclones.

2.3.2 Proof of concept: exemplary application of our estimation approach

To visualize clonal evolution in our study, we use the R package "fishplot". Due to the presence of few time points, we choose to apply an estimation approach leading to improved visualization. To prove that our approach does not lead to any alternations in the basic results, we consider clonal evolution in 11 patients with MDS, published by da Silva-Coelho *et al.* 2017 [1]. For every sample, data on 5 to 30 time points is available.

For every sample, information on VAF of the different mutations and the assigned clones is available. We estimate cell ratios for the different (sub)clones based on the average VAF over all mutations defining a (sub)clone. Following this approach, we have to exclude patients UPN02, UPN05 and UPN10 from further consideration. UPN02 shows VAFs>50% for TET2 mutation, despite no CNV being reported on chromosome 4. UPN05 is excluded as the percentage of cells for dependent subclones (purple and orange) sum up to more than the percentage of their parent (red). UPN10 is excluded as percentage of cells with the dark green clone, being only characterized by a deletion of 13q, are just available at one time point. Additionally, UPN04 was excluded as this patient shows basically no clonal evolution, featuring only one clone and no subclones.

We split the remaining patients into 2 subgroups – just like in case of of the publication by da Silva-Coelho *et al.* 2017: 1) patients who received supportive care only (UPN3, UPN06, UPN07, UPN11), 2) patients who were treated with lenalidomide (UPN01, UPN08, UPN09). For both subgroups, we keep only the very first and very last time point. Subsequently, we estimate additional time points, following the procedure described in sections 1.9.2 and 1.9.3.

For subgroup 1, we apply our 1st step of estimating additional time points only. The results, comparing clonal evolution based on all measured time points vs 2 time points plus additional estimated time points, are visualized in Figure S13.

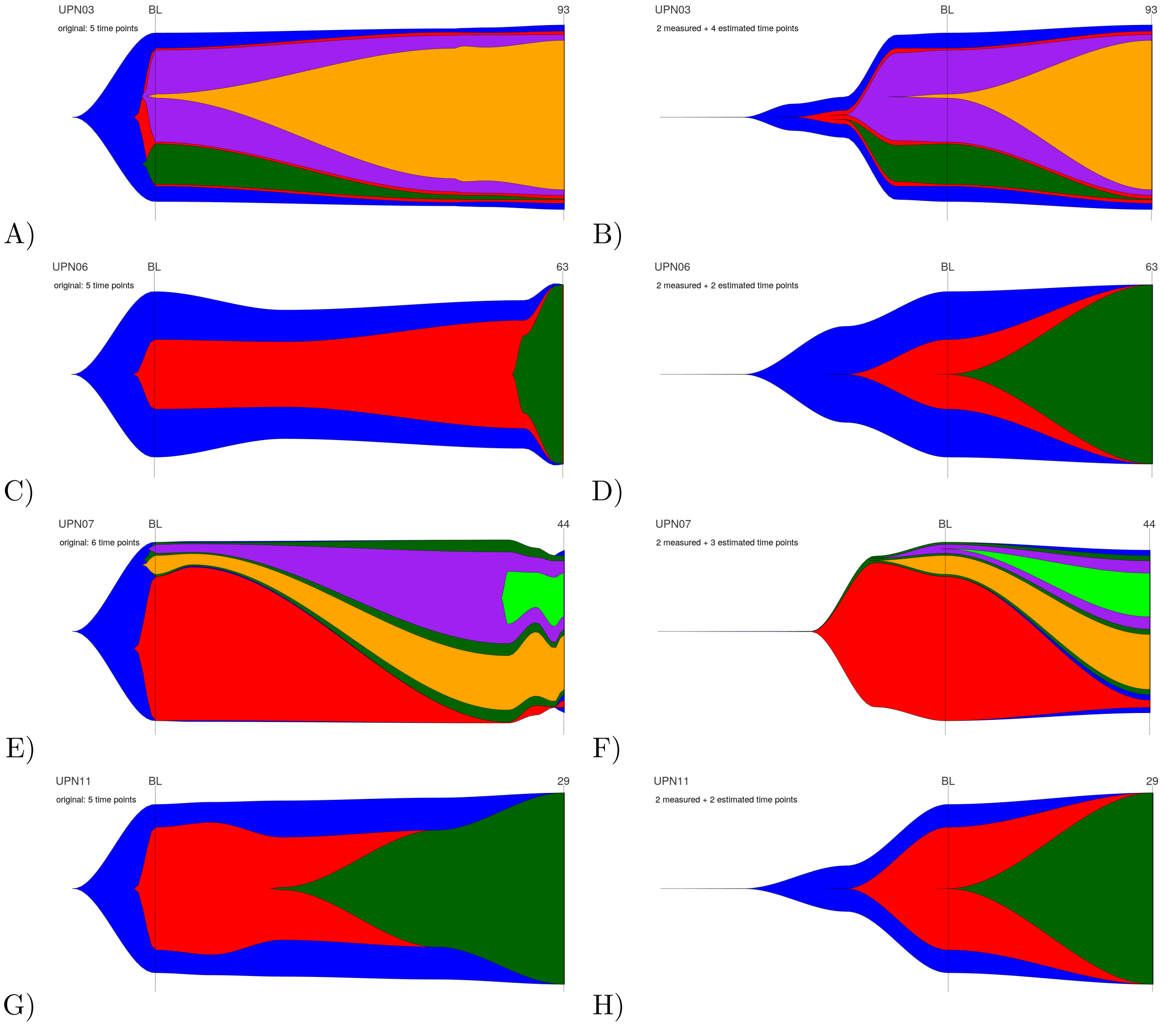

Figure S13: Estimated and real clonal evolution in MDS. Clonal evolution for MDS patients UPN03, UPN06, UPN07 and UPN11. A, C, E, G) Evaluating all measured time points. B, D, F, H) Evaluating the first and last measured time points and adding additional estimated time points.

For subgroup 2, we apply our whole 2-step procedure of estimating additional time points, including estimation of therapy effect. The results, comparing clonal evolution based on all measured time points vs 2 time points plus additional estimated time points, are visualized in Figure S14.

In both Figures S13 and S14 it can be observed that clonal evolution considering only 2 time points – the very first and last – and additional estimated time points, matches the original plots quite well. Even in case of sample UPN01, where clonal evolution is originally based on 15 time points, our approach reflects the actual development of clones well.

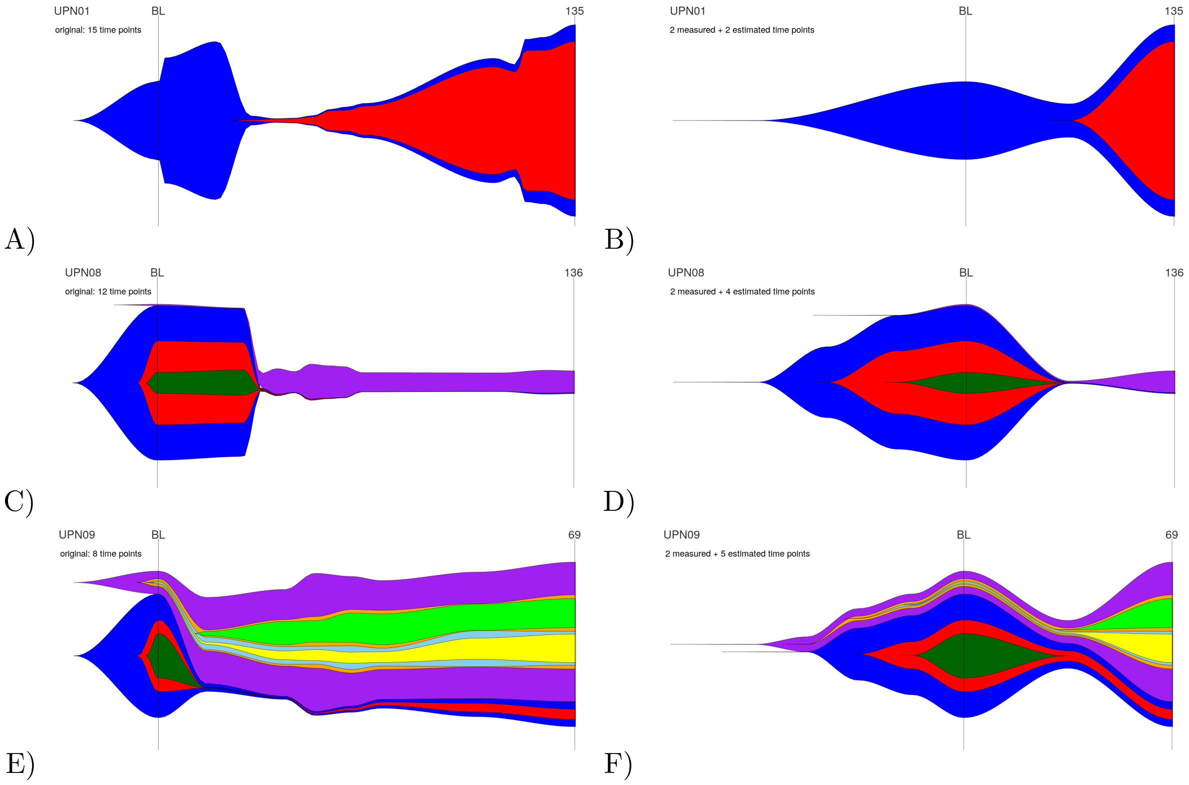

Figure S14: Estimated and real clonal evolution in MDS including therapy effect. Clonal evolution for MDS patients UPN01, UPN08 and UPN09. A, C, E) Evaluating all measured time points. B, D, F) Evaluating the first and last measured time points and adding additional estimated time points.

Concluding from these analyses, we assume that our step 1 – estimating development of clones over time – successfully estimates clonal evolution at additional time points, where no sequencing has been performed. Furthermore, we assume that step 2 – estimating effect of therapy – is able to approximate the effect of therapy if, like in case of our study, not just supportive care was provided.

2.3.3 Clonal evolution without estimated time points

The reconstructed clonal evolution solely based on the measured time points is visible in Figure S15 for all relapse patients.

It can be observed that several subclones and their evolution over time are hard to identify. At time point 1, identification of the different subclones and their percentage of cells in tumor is difficult. This is especially true for patients with complex clonal evolution, including many subclones, e.g. patient 5 (Figure S15 E). At time point 2, it is no longer possible – except for patient 3 – to visualize any subclones that have not been present at time point 1 (compare e.g. Figure S15 A to Figure 2 A for patient 1). Furthermore, likely effect of therapy on clonal evolution is not visible.

2.3.4 Automatically reconstructed clonal evolution

In addition to manual reconstruction of clonal evolution, we also applied tools for automatic reconstruction. The results for PyClone in combination with ClonEvol, in comparison to our manual approach, are summed up in table S9.

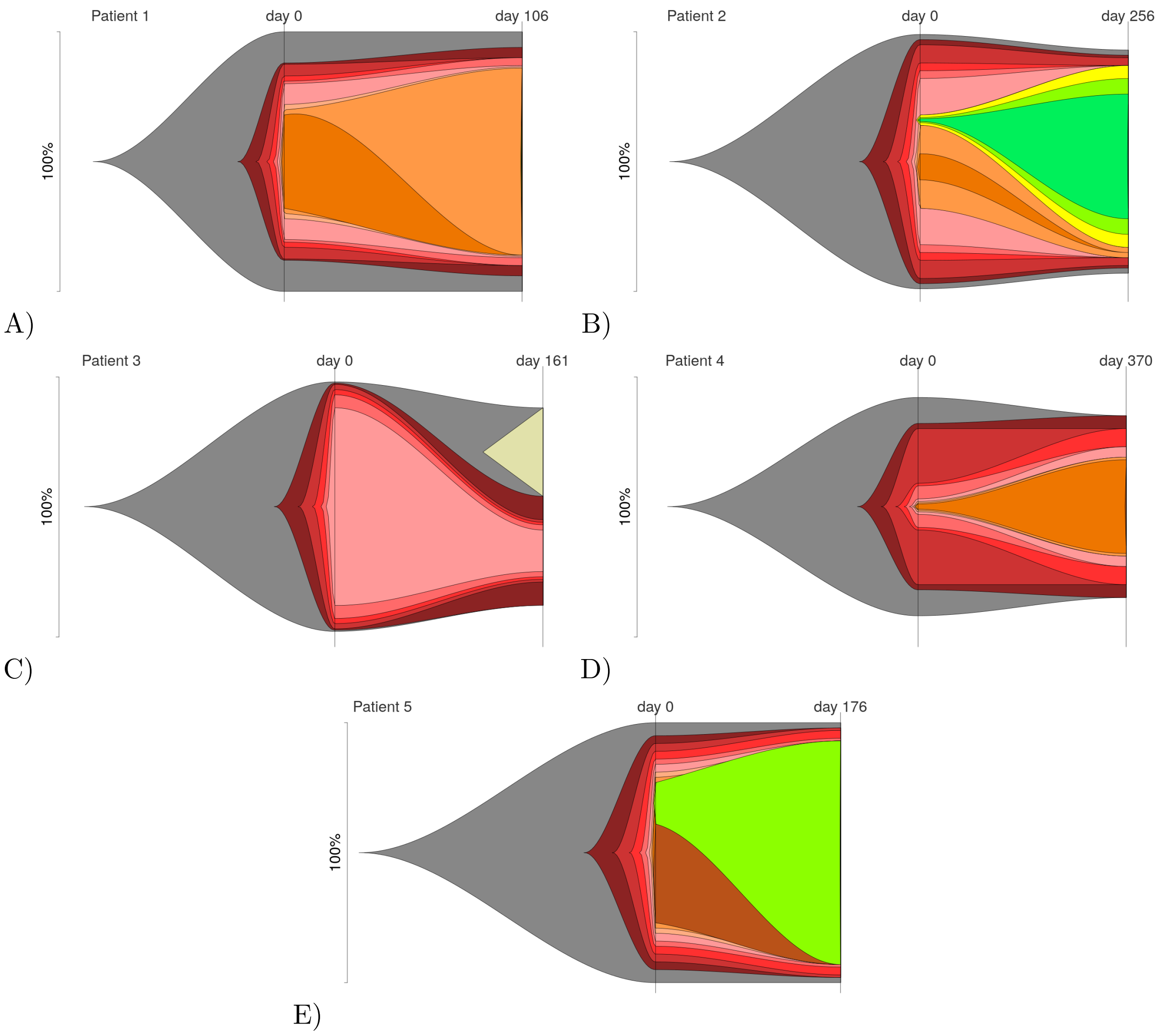

Figure S15: Alternative clonal evolution plots for relapse patients 1-5. The plots show just the 2 measured time points and no estimated development in between. A) Patient 1. B) Patient 2. C) Patient 3. D) Patient 4. E) Patient 5.

Table S9: Clonal evolution with PyClone and ClonEvol. Automatically reconstructed clonal evolution, using PyClone in combination with ClonEvol, in comparison to manually reconstructed clonal evolution.

Patient	Manual reconstruction		Automatic reconstruction	
	Cluster	Clonal evolution type	PyClone cluster	ClonEvol evolution type
1	17	Parallel dependent: gained dominance	6	no consensus model
2	13	Parallel dependent: gained dominance	8	no consensus model
3	7	Parallel dependent: gained co-existence	3	no possible founding cluster
4	10	Linear	4	Linear
5	17	Parallel dependent: gained dominance	11	no possible founding cluster

Taking the information on `cluster_id` and `cellular_prevalence` for every mutation (as reported by PyClone in `loci.tsv`) as input, ClonEvol is applied in CCF-mode (defining `ccf.col.names`). However, just in case of one sample a consensus model could be reconstructed. PyClone itself did not succeed in reconstructing any plots based on the input data. To examine the source of error, we manually inspected the PyClone output for every sample. For patients 1, 2 and 5, no clonal evolution fits the automatically determined cluster and their cancer cell

fractions. For patient 3, a linear clonal evolution model may be reconstructed from PyClone output, if deviations from the reported cancer cell fraction by ≥ 1 standard deviation are accepted. However, as PyClone could not consider the only relapse-specific variants (two CNVs), as well as 19 CNVs already present at primary diagnosis, it is likely that this linear model does not reflect actual clonal evolution in patient 3.

For patient 4, clonal evolution could automatically be reconstructed. PyClone and ClonEvol indicate a linear evolution just like we also reconstructed manually. The models only differ in the number of clusters: Clones 1 to 3 are summed up to just one cluster by PyClone. Results for clone 4 do not differ. Clones 5 to 10 are summed up to just two clusters by PyClone. The first cluster contains mutations with VAFs between 1.89% and 7.98% at primary diagnosis and 16.42% and 28.29% at relapse. The second cluster contains mutations with VAFs between 0% and 0.83% at primary diagnosis and 15.34% and 30.00% at relapse. Due to overlapping VAF-ranges, it appears likely that clustering by PyClone is too rough. However, as we just have two time points and coverage is in some regions <50x, a final conclusion on which clustering is correct, cannot be drawn. It should be noted that clonal evolution reconstructed by PyClone does not contain the 3 relapse-specific CNVs detected for patient 4.

To investigate robustness of the tools' automatically reconstructing clonal evolution, we considered a second approach combining SciClone with ClonEvol. The results, in comparison to our manual approach, are summed up in table S10.

Table S10: Clonal evolution with SciClone and ClonEvol. Automatically reconstructed clonal evolution, using SciClone in combination with ClonEvol, in comparison to manually reconstructed clonal evolution. Application of ClonEvol is only possible in the presence of >1 time point, i.e. not for the non-relapse samples.

Patient	Manual reconstruction		Automatic reconstruction		
	Cluster	Clonal evolution type	SciClone cluster	Ignored mutations	ClonEvol evolution type
1	17	Parallel dependent: gained dominance	4	53/112 (47%)	Linear
2	13	Parallel dependent: gained dominance	2	13/29 (45%)	Parallel dependent: gained dominance
3	7	Parallel dependent: gained co-existence	clustering failed		/
4	10	Linear	clustering failed		/
5	17	Parallel dependent: gained dominance	1	17/41 (42%)	no evolution

Taking the information on `cluster`, `Sample1a.vaf` and `Sample1b.vaf` for every mutation (as reported by SciClone in `clusters2`) as input, ClonEvol is applied in VAF-mode (defining `vaf.col.names`). However, results could only be generated for two patients. Clustering with SciClone failed for patients 3 and 4. For patient 5, only 1 cluster was determined by SciClone, which is why no clonal evolution tree could be calculated by ClonEvol.

For patient 1, it is possible to reconstruct clonal evolution automatically. Linear evolution is predicted. However, SciClone clusters only 53% of all variants, while ignoring the others. According to documentation, these mutations were removed because of copy-number alterations or inadequate depth. Additionally, no cluster information is reported for any copy number variant. Thus, it appears unlikely that this clonal evolution, considering only half of the available information, is closer to reality compared to our manually reconstructed evolution, considering all available information.

For patient 2, it is also possible to reconstruct clonal evolution automatically. Parallel dependent evolution with gained dominance is predicted, which is the same type of evolution as we reconstructed manually. Comparing the results, our manual reconstruction contains much more clusters. However, SciClone ignores 45% of all mutations and all 9 CNVs. Thus, it appears likely that the true clonal evolution is characterized by more than just 1 clone plus 1 subclone.

2.4 Supplementary Data

Data S1: SupplementaryData_1.xlsx: SNV and indel calling results for 10 cases of sporadic Burkitt lymphoma, including 5 cases with relapse. (A) Somatic SNV and indel calls based on matched whole-exome sequencing data analysis reporting chromosome, position, reference, alternative, gene, exon, transcript ID, type, mutation, codon change, read counts and VAF for tumor sample, read counts and VAF for germline sample. (B) True positive validation results based on targeted sequencing and Sanger sequencing. (C) True negative variant calls. Manual inspection categorized these calls as artifacts. The categorization could be validated by targeted sequencing. (D) False negative variant calls. Common hotspot mutations were investigated by Sanger sequencing for both samples with and without variant calls in WES data. Results indicate no false negative variant calls.

Data S2: SupplementaryData_2.xlsx: CNV calling results for 10 cases of sporadic Burkitt lymphoma, including 5 cases with relapse. (A) CNV calls based on SNP array data analysis reporting chromosome, start position, end position, CNV value and type. Rate of cells (considering mean and standard deviation) affected by CNV is estimated on the basis of SNP array data, WES data and WES data with extended regions.

Data S3: SupplementaryData_3.xlsx: Combined SNV, indel and CNV results forming the basis for reconstruction of clonal evolution. Mutations are clustered according to (sub)clones. (A) SNV, indel and CNV results for patient 1, reporting chromosome, position, reference, alternative, gene, type, redcounts and VAF for whole-exome sequencing germline, primary and relapse sample, clone, parent clone. Information on (sub)clones is colored according to colors used in clonal evolution plots. SNVs and indels affected by CNVs, and sex chromosomes are colored in grey. (B) Patient 2. (C) Patient 3. (D) Patient 4. (E) Patient 5. (F) Patient 6. (G) Patient 7. (H) Patient 8. (I) Patient 9. (J) Patient 10.

References

[1] da Silva-Coelho P, Kroeze LI, Yoshida K, Koorenhof-Scheele TN, Knops R, van de Locht LT, et al. Clonal evolution in myelodysplastic syndromes. *Nat Commun.* 2017;**8**(15099).

[2] Woesmann W, Seidemann K, Mann G, Zimmermann M, Burkhardt B, Oschlies I, et al. The impact of the methotrexate administration schedule and dose in the treatment of children and adolescents with B-cell neoplasms: a report of the BFM Group Study NHL-BFM95. *Blood.* 2005;**105**(3):948–958.

[3] Li H, Durbin R. Fast and accurate short read alignment with Burrows-Wheeler transform. *Bioinformatics.* 2009;**25**(14):1754–1760.

[4] Larson DE, Harris CC, Chen K, Koboldt DC, Abbott TE, Dooling DJ, et al. SomaticSniper: identification of somatic point mutations in whole genome sequencing data. *Bioinformatics.* 2012;**28**(3):311–317.

[5] Saunders CT, Wong WS, Swamy S, Becq J, Murray LJ, Cheetham RK. Strelka: accurate somatic small-variant calling from sequenced tumor-normal sample pairs. *Bioinformatics.* 2012;**28**(14):1811–1817.

[6] Koboldt DC, Zhang Q, Larson DE, Shen D, McLellan MD, Lin L, et al. VarScan 2: Somatic mutation and copy number alteration discovery in cancer by exome sequencing. *Genome Res.* 2012;**22**(3):568–576.

[7] Cibulskis K, Lawrence MS, Carter SL, Sivachenko A, Jaffe D, Sougnez C, et al. Sensitive detection of somatic point mutations in impure and heterogeneous cancer samples. *Nat Biotechnol.* 2013;**31**(3):213–219.

[8] Sandmann S, Karimi M, de Graaf AO, Rohde C, Göllner S, Varghese J, et al. appreci8: a pipeline for precise variant calling integrating 8 tools. *Bioinformatics.* 2018;**34**(24):4205–4212.

[9] Cingolani P, Platts A, Wang le L, Coon M, Nguyen T, Wang L, et al. A program for annotating and predicting the effects of single nucleotide polymorphisms, SnpEff: SNPs in the genome of Drosophila melanogaster strain w1118; iso-2; iso-3. *Fly.* 2012;**6**(2):80–92.

[10] Robinson JT, Thorvaldsdóttir H, Winckler W, Guttman M, Lander ES, Getz G, et al. Integrative genomics viewer. *Nat Biotechnol.* 2011;**29**(1):24–26.

[11] Consortium TGP. A global reference for human genetic variation. *Nature.* 2015;**526**(7571):68–74.

[12] Lek M, Karczewski KJ, Minikel EV, Samocha KE, Banks E, Fennell T, et al. Analysis of protein-coding genetic variation in 60,706 humans. *Nature.* 2016;**536**(7616):285–291.

[13] Sherry ST, Ward MH, Kholodov M, Baker J, Phan L, Smigielski EM, et al. DbSNP: the NCBI database of genetic variation. *Nucleic Acids Res.* 2001;**29**(1):308–311.

[14] Landrum MJ, Lee JM, Benson M, Brown G, Chao C, Chitipiralla S, et al. ClinVar: public archive of interpretations of clinically relevant variants. *Nucleic Acids Res.* 2016;**44**(D1):D862–D868.

[15] Forbes SA, Beare D, Gunasekaran P, Leung K, Bindal N, Boutselakis H, et al. COSMIC: exploring the world's knowledge of somatic mutations in human cancer. *Nucleic Acids Res.* 2015;**43**(Database issue):D805–D811.

[16] Sanger F, Nicklen S, Coulson AR. DNA sequencing with chain-terminating inhibitors. *Proc Natl Acad Sci U S A*. 1977;**74**:5463–5467.

[17] Lawrence MS, Stojanov P, Polak P, V KG, Cibulskis K, Sivachenko A, et al. Mutational heterogeneity in cancer and the search for new cancer-associated genes. *Nature*. 2013;**499**:214–218.

[18] Stelzer G, Plaschkes I, Zimmermann S, *et al* . The GenCards Suite: From Gene Data Mining to Disease Genome Sequence Analyses. *Current Protocols in Bioinformatics*. 2016;**54**(1):1.30.1–1.30.33.

[19] Miller CA, McMichael J, Dang HX, Maher CA, Ding L, Ley TJ, et al. PaxtoolsR: pathway analysis in R using Pathway Commons. *Bioinformatics*. 2016;**32**(8):1262–1264.

[20] Miller CA, McMichael J, Dang HX, Maher CA, Ding L, Ley TJ, et al. Visualizing tumor evolution with the fishplot package for R. *BMC Genomics*. 2016;**17**:880.

[21] Roth A, Khattra J, Yap D, Wan A, Laks E, Biele J, et al. PyClone: statistical inference of clonal population structure in cancer. *Nat Methods*. 2014;**4**:396–398.

[22] Miller CA, White BS, Dees ND, Griffith M, Welch JS, Griffith OL, et al. SciClone: Inferring Clonal Architecture and Tracking the Spatial and Temporal Patterns of Tumor Evolution. *PLoS Comput Biol*. 2014;**10**(8):e1003665.

[23] Dang HX, White BS, Foltz SM, Miller CA, Luo J, Fields RC, et al. ClonEvol: clonal ordering and visualization in cancer sequencing. *Ann Oncol*. 2017;**28**(12):3076–3082.

[24] Huang DW, Sherman BT, Lempicki RA. Bioinformatics enrichment tools: paths toward the comprehensive functional analysis of large gene lists. *Nucleic Acids Res*. 2009;**37**(1):1–13.

[25] Huang DW, Sherman BT, Lempicki RA. Systematic and integrative analysis of large gene lists using DAVID Bioinformatics Resources. *Nat Protoc*. 2009;**4**(1):44–57.

[26] Rohde M, Bonn BR, Zimmermann M, Lange J, Möricke A, Klapper W, et al. Relevance of ID3-TCF3-CCND3 pathway mutations in pediatric aggressive B-cell lymphoma treated according to the non-Hodgkin lymphoma Berlin-Frankfurt-münster protocols. *Haematologica*. 2017;**102**(6):1091–1098.

[27] Bouska A, Bi C, Lone W, Zhang W, Kedwaii A, Heavican T, et al. Adult high-grade B-cell lymphoma with Burkitt lymphoma signature: Genomic features and potential therapeutic targets. *Blood*. 2017;**130**(16):1819–1831.

[28] Boguslawska J, Sokol E, Rybicka B, Czubaty A, Rodzik K, Piekielko-Witkowska A. microRNAs target SRSF7 splicing factor to modulate the expression of osteopontin splice variants in renal cancer cells. *Gene*. 2016;**595**(2):142–149.

[29] Lee J, Beliakoff J, Sun Z. The novel PIAS-like protein hZimp10 is a transcriptional coactivator of the p53 tumor suppressor. *Nucleic Acids Res*. 2007;**35**(13):4523–4534.

[30] Matsumoto M, Minami M, Takeda K, Sakao Y, Akira S. Extopic expression of CHOP (GADD153) induces apoptosis in M1 myeloblastic leukemia cells. *FEBS Lett*. 1996;**395**(1-3):143–147.

[31] Vu LP, Prieto C, Amin EM, Chhangawala S, Krivtsov A, Calvo-Vidal MN, et al. Functional screen of MSI2 interactors identifies an essential role for SYNCRIP in myeloid leukemia stem cells. *Nat Genet*. 2017;**49**(6):866–875.

[32] Yang L, Zhang H, Yao Q, Wu R, Liu M. Clinical significance of SASH1 Expression in Glioma. *Dis Markers.* 2015;.

[33] Adzhubei IA, Schmidt S, Peshkin L, Ramensky VE, Gerasimova A, Bork P, et al. A method and server for predicting damaging missense mutations. *Nat Methods.* 2010;**7**(4):248–249.

[34] Forero-Castro M, Robledo C, Lumbreras E, Benito R, Hernández-Sánchez JM, Hernández-Sánchez MG, et al. The presence of genomic imbalances is associated with poor outcome in patients with burkitt lymphoma treated with dose-intensive chemotherapy including rituximab. *Br J of Haematol.* 2016;**172**(3):428–438.
